# A Two-Locus Model of the Evolution of Insecticide Resistance to Inform and Optimise Public Health Insecticide Deployment Strategies

**DOI:** 10.1371/journal.pcbi.1005327

**Published:** 2017-01-17

**Authors:** Bethany Levick, Andy South, Ian M. Hastings

**Affiliations:** 1 Department of Parasitology, Liverpool School of Tropical Medicine, Liverpool, United Kingdom; 2 Independent consultant, Norwich, United Kingdom; University of Chicago, UNITED STATES

## Abstract

We develop a flexible, two-locus model for the spread of insecticide resistance applicable to mosquito species that transmit human diseases such as malaria. The model allows differential exposure of males and females, allows them to encounter high or low concentrations of insecticide, and allows selection pressures and dominance values to differ depending on the concentration of insecticide encountered. We demonstrate its application by investigating the relative merits of sequential use of insecticides versus their deployment as a mixture to minimise the spread of resistance. We recover previously published results as subsets of this model and conduct a sensitivity analysis over an extensive parameter space to identify what circumstances favour mixtures over sequences. Both strategies lasted more than 500 mosquito generations (or about 40 years) in 24% of runs, while in those runs where resistance had spread to high levels by 500 generations, 56% favoured sequential use and 44% favoured mixtures. Mixtures are favoured when insecticide effectiveness (their ability to kill homozygous susceptible mosquitoes) is high and exposure (the proportion of mosquitoes that encounter the insecticide) is low. If insecticides do not reliably kill homozygous sensitive genotypes, it is likely that sequential deployment will be a more robust strategy. Resistance to an insecticide always spreads slower if that insecticide is used in a mixture although this may be insufficient to outperform sequential use: for example, a mixture may last 5 years while the two insecticides deployed individually may last 3 and 4 years giving an overall ‘lifespan’ of 7 years for sequential use. We emphasise that this paper is primarily about designing and implementing a flexible modelling strategy to investigate the spread of insecticide resistance in vector populations and demonstrate how our model can identify vector control strategies most likely to minimise the spread of insecticide resistance.

## Author Summary

Malaria results in hundreds of thousands of deaths annually. The two most effective malaria control interventions have been insecticide treated bed nets and indoor residual spraying of insecticides but their successes are now threatened by insecticide resistance in mosquitoes. Insecticide deployment strategies can be designed to try to minimise the spread of insecticide resistance. We develop a highly flexible model of the evolution of insecticide resistance which we use to assess the relative performance of two insecticide deployment strategies: two insecticides used in sequence (where one is replaced by the other once a resistance threshold is reached) or as a mixture (where they are applied together). The relative performance of the strategies depended on the input parameter values that are likely to depend on the insecticide properties and local malaria transmission patterns. In the parameter space we explored, mixtures were favoured when insecticide effectiveness (ability to kill wildtype susceptible mosquitoes) was high and exposure (the proportion of mosquitoes that encounter the insecticide) was low. If insecticides do not reliably kill susceptible mosquitoes, it is likely that sequential deployment will be better. This flexible model is designed to enable detailed exploration of deployment issues and their influence on the strategies most likely to minimise the evolution of insecticide resistance.

## Introduction

Insect populations are controlled for many reasons: as agricultural pests, to reduce nuisance-biting mosquitoes, and to control a variety of vector-borne infectious diseases. The World Health Organisation (WHO) estimates that 17% of all infectious diseases are vector-borne [1], important examples being malaria, dengue, Japanese encephalitis, filariasis and, most recently, Zika virus. This manuscript will focus on the control of mosquitoes responsible for transmitting human malaria, which is the target of considerable public health efforts through deployment of insecticide-treated bed nets (ITNs) and insecticides sprayed onto house walls (indoor residual spraying; IRS). Bed nets and IRS both require persistent, long-acting insecticides and this combination of persistence and widespread coverage acts as a potent driver of insecticide resistance [2]. The evolution of insecticide resistance (IR) appears to be a near-inevitable consequence of attempting to control insect populations through insecticides and there is considerable interest in designing and optimising strategies to offset its impact. Species of anopheline mosquitoes in Africa (where malaria kills close to 0.4 million people per year) are becoming increasing resistant to the insecticides used in personal protection as ITNs and in public health campaigns based on IRS [2]. Recent estimates are that ITNs and IRS were responsible for 68% and 13%, respectively, of the substantial declines in falciparum malaria clinical cases observed in Africa between 2000 and 2015 [3]. Concerns over the potential impact of IR has led the WHO to produce comprehensive plans to try and maintain their effectiveness in malaria control [4] and the influential Insecticide Resistance Action Committee (IRAC; http://www.irac-online.org/) has produced similar, but more general guidelines for controlling vectors of other human vector-borne disease [5].

There are four main strategies that may potentially be used as insecticide deployment strategies. Sequential use, with each insecticide being replaced as the resistance arises to the previous one. This has been the *de facto* strategy in public health where pyrethroids have been the major insecticides used to date due to their low cost and low human toxicity (they are the only class able to be used on ITNs). Mixtures constitute an alternative strategy where insecticides are co-deployed as mixtures of two or more compounds. The reasoning underlying mixtures is that using two different insecticides means that mosquitoes must be resistant to both insecticides to survive. Assuming independent mechanisms of resistance, resistance mutations must then be present at two different genes to survive contact with a mixture, potentially slowing the evolution of IR. There is considerable interest in mixtures as it is now mandatory to use drugs in combination to control resistance in the main human infectious killers of the tropics i.e. malaria, HIV and TB. Theoretical descriptions of this are well established for malaria parasites [6], HIV [7] and TB (e.g. [8]) but is surprisingly poorly developed for IR in human disease vectors. Rotations are a strategy where one insecticide is periodically withdrawn from use and replaced by another, irrespective of its resistance profile; this has been used in agriculture but not, to our knowledge, in public health. Mosaics are the final option, where different insecticides are deployed alone but in different settings. The mosaics may differ in scale: it may be that ITN and IRS use different insecticides in the same house, it may be that adjacent villages use different insecticides in their IRS programmes, or it may be that different health districts in the same country use different insecticides. There was a pilot study of mosaics [9] but this has not been adopted as policy. Much of the deployment in public health has been rather *ad hoc* and driven primarily by cost and availability in their typical, resource poor settings. DDT was widely used until environmental concerns plus increasing resistance curtailed its used. Pyrethroids are now the mainstay due to low cost and low toxicity. Carbamates may be used as IRS but are expensive. The lack of insecticide options and increasing levels of resistance stimulated the Bill and Melinda Gates Foundation to support an Innovative Vector Control Consortium (IVCC) [10] whose remit was to develop and/or re-purpose insecticides for public health use. They now have three products nearing the end of their R&D pipeline and nearly ready for deployment. There is consequently an excellent opportunity to deploy these new products in a planned, rational way to minimise selection for resistance while maintaining their impact in disease control; the key policy question is to identify the best way(s) to achieve this goal.

There is considerable literature on how insecticide deployment policies drive, or may avert, the evolution of IR (or the analogous example of genetically-encoded insecticidal toxins) in agriculture (e.g. [11–16]). This work has been relatively well resourced either by agrichemical companies directly, or by Western governments where IR imposes a substantial economic cost on their agricultural sector. Much of that work is relevant to public health in the tropics but there are two important differences. Firstly, the insect species differ in their life histories: vectors must bite humans and often it is only the females which feed. There is often considerable sex-differences in insecticide exposure that typically do not occur in agricultural pest species. A second difference is in the degree of control the authorities have over insecticide deployment. Agricultural deployment, at least in developed countries, may be controlled by regulatory bodies and/or the agro-chemical industries and/or farming organisations. In contrast, in public health there are severe operational difficulties that dictate that robust, simple polices be implemented. For example, insecticides in agricultural use are often deployed as rotation where one insecticide is periodically replaced by another irrespective of resistance status. Such strategies might be desirable in tropical public health but is difficult to see how this could be implemented in the face of challenges arising from relative costs, the need to retrain operators, maintenance of robust supply-chains and so on. Consequently, the literature on agricultural use has developed in a rather independent manner from that applied to public health. The best known example of the latter was written by the late Chris Curtis and published in 1985 [17]. This has been highly influential. It has been cited nearly 200 times, not just for IR, but for analogous situations of drug resistance in other sexual diploid organisms such as parasitic worms. Curtis explored the application of his model to mosquito vectors of malaria with particular emphasis on policy implications. One key finding reported in that paper was the “at first sight unexpected, point that the use of a mixture where the initial gene frequencies are unequal leads to more rapid increase in the frequency of the rarer of the genes”. Since any new insecticide becoming available for the public health deployment would almost inevitably be the insecticide to which resistance was “rarer”, this paper implies that any new insecticide should not be used in a mixture. Our results (see later) suggest this statement is open to a different interpretation but this assertion has influenced public-health insecticide deployment policy for nearly 30 years.

**Fig 1:**
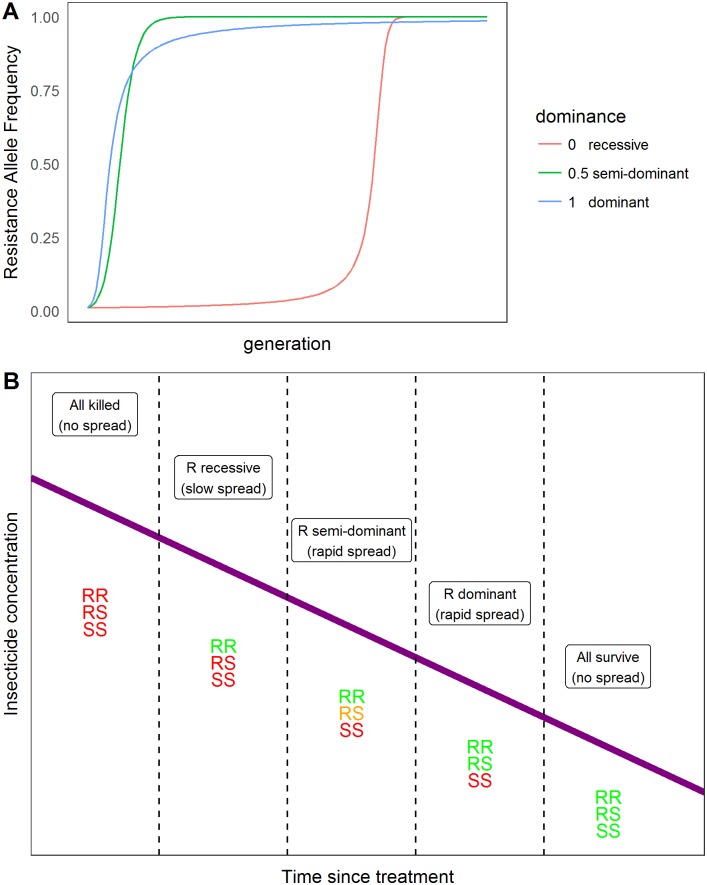
The importance of genetic dominance and the need for different ‘niches’ in the model. (A) The rate of spread of insecticide resistance depending on whether the resistance mutation is dominant, semi-dominant, or recessive. (B) An illustration showing how the concentration of insecticide alters selection for resistance and the dominance level of resistance alleles. Declining concentration of insecticide resistance is represented by the purple line and the colour of the genotypes indicates whether they survive (green), die (red) or have borderline sensitivity (yellow) at that insecticide concentration. Initially, insecticide concentration may be so high that no genotypes survive and there is consequently no selection for resistance; note that this stage may not occur if resistance alleles encode very high levels of resistance and/or if the insecticide is applied at sub-optimal concentrations. As concentrations decline, they start to select for resistance. At relatively high concentrations only the RR individuals survive so resistance is recessive; as concentrations decline further some RS mosquitoes survive making resistance semi-dominant and at low concentration both RR and RS mosquitoes survive, making resistance dominant; at very low concentrations all three genotype survive and there is no further selection for resistance (for further discussion see, for example, [20, 21]).

A key tenet of science is that results should be repeatable. Unfortunately, Curtis did not publish the equations used to implement his model, nor has it been possible to find the computer code among his personal documents and computer file that remain in the London School of Hygiene and Tropical Medicine (J. Lines, pers. Comm.). In addition, the paper contained results based on both single-generation and multi-generation arguments. Consequently, it is impossible at present to easily duplicate his work nor to re-calibrate and re-run his models to assess whether his assertions are robust across all parameter values. This manuscript describes how we developed a model to repeat and extend his results in an open-source framework following modern good practice for developing scientific software (e.g. [18]). The Curtis model also made several assumptions, presumably dictated by computing restrictions at the time that we will relax in the development of our current model. Specifically:

- Curtis assumed that male and female mosquitoes are equally exposed to insecticide. The latter is problematic for Anopheles mosquitoes because only females bite humans and are consequently believed to be more highly exposed to insecticides in those species that forage into human habitation to find hosts (e.g. [19]). We therefore allowed differential exposure of males and females to insecticide.
- We also allowed a more flexible description of how mosquitoes encounter insecticides. One of the key determinants of how quickly IR evolves is the extent to which resistance mutations are genetically dominant. The degree of dominance can change depending on the level of insecticide that is encountered (e.g. [20, 21]), see Fig 1. We therefore allow mosquitoes to encounter none/low/high concentrations of each of two insecticides (although time constraints precluded us exploring this option). This leads to 9 distinct ‘niches’ and the user can define differential exposure of males and females to these niches.

The basic model construction using ‘niches’ is shown on Table 1. The design principle was to make the model as flexible as possible with the intention of allowing specific scenarios to be investigated as simpler subsets of the models. For example, the Curtis model will be recreated by only allowing potential exposure in four niches (no insecticide, high concentration of insecticide ‘A’, high concentration of insecticide ‘B’, a mixture of ‘A’ and ‘B’) and to set equal male/female exposure within these niches. The current model design is flexible as it allows us to investigate some key operational scenarios e.g. the consequences of not regularly spraying walls or replacing bednets so that the ‘high concentration mixture’ niche is gradually replaced by ‘Low concentration mixture niche’ with, potentially, a corresponding change in dominance. Similarly, we can investigate the simultaneous co-deployment of both mixtures and single-insecticides, any putative repellency of insecticides driving more mosquitoes into the unexposed niche [19], and so on.

**Table 1:** The basic model construction. Two insecticides are deployed i.e. “a/A” and “b/B” each of which may be absent (symbol “-“), present in low concentrations (lower case “a” or “b”) or present at high concentrations (upper case “A” or “B”). This gives nine insecticide-exposure “niches” in total, one in which insecticide is completely absent, four in which a single insecticide is present alone, and four where both insecticides are present. The model allows differential exposure patterns of males and females to the niches; their exposure is quantified as α with the superscript (m, f) denoting the sex of the mosquito and the subscript denoting the niche. These α values are the proportions encountering each niche so that, obviously, the male α values must sum to unity, as must the female exposures.

	Insecticide niche								
Insecticide encountered	-, -	a, -	A, -	b, -	B, -	a, b	A, b	a, B	A, B
*Male exposure*	$\alpha_{-,-}^{m}$	$\alpha_{a,-}^{m}$	$\alpha_{A,-}^{m}$	$\alpha_{b,-}^{m}$	$\alpha_{B,-}^{m}$	$\alpha_{a,b}^{m}$	$\alpha_{A,b}^{m}$	$\alpha_{a,B}^{m}$	$\alpha_{A,B}^{m}$
*Female exposure*	$\alpha_{-,-}^{f}$	$\alpha_{a,-}^{f}$	$\alpha_{A,-}^{f}$	$\alpha_{b,-}^{f}$	$\alpha_{B,-}^{f}$	$\alpha_{a,b}^{f}$	$\alpha_{A,b}^{f}$	$\alpha_{a,B}^{f}$	$\alpha_{A,B}^{f}$

## Methods

The methodology and underlying assumptions made in deriving the methodology are standard ones generally made in the construction of population genetics models (e.g. [22, 23]; we assume that many readers will be unfamiliar with the methodologies so discuss them in more detail than is usual in genetic publications.

### Model construction and initial calibration

The first step is to define the exposure of males and females to each of the nine niches as defined on Table 1. These values lie between 0 and 1, representing the proportion of the population likely to contact that niche. Individual niches can be ignored simply by setting their exposures to zero.

The second step is to define the fitness of the different genotypes (Table 2); these are all scaled relative to the fully sensitive mosquito in the absence of insecticide (as in a previous single-locus model of resistance evolution [24]) whose fitness is denoted 1. ‘Fitness’ is essentially a measure of the reduced viability (i.e. death rate) following contact with the insecticide), and/or of decreased fertility (i.e. mosquitoes surviving contact with an insecticide subsequently produce fewer viable offspring). The population genetic methodology means it is unnecessary to define the mechanism by which fitness is reduced.

**Table 2:** Model calibration. This follows conventional population genetic methodology and is achieved by assigning the fitness of each genotype at each locus, assuming that Locus 1 encodes resistance to insecticide A and Locus 2 encodes resistance to insecticide B. The symbols ‘w’ define the genotype fitnesses whose superscripts indicate the genotype (SS, RS, RR) and the locus at which it is located (1, 2) and whose subscripts (-/a/A/b/B) indicate the niche in which fitness is defined where ‘-‘ is insecticide-free and a/A and b/B indicate the presence of insecticides a/A or b/B respectively, in high (upper case) or low (lower case) concentration,. These fitnesses are determined by the effectiveness of the insecticides φ, (i.e. the proportion of SS genotypes killed after contact with the insecticide); setting φ <1 allows a proportion of SS genotypes to survive contact with the insecticide, h is the dominance coefficient, s is selection coefficient favouring IR, and z is fitness cost of carrying resistance alleles in the absence of insecticide.

Genotype	Locus	Insecticide present				
		-	a	A	b	B
**SS**	**1**	$w_{-}^{SS1}\;=\;1$	$w_{a}^{SS1}\;=\;1-\varphi_{a}^{SS1}$	$w_{A}^{SS1}\;=\;1-\varphi_{A}^{SS1}$		
**RS**	**1**	$w_{-}^{RS1}\;=\;1-h_{-}^{1}{\;z}_{-}^{RR1}$	$w_{a}^{RS1\;}\;=\;w_{a}^{SS1\;}+\;h_{a}^{}s_{a}^{RR1}$	$w_{A}^{RS1\;}\;=\;w_{A}^{SS1\;}+\;h_{A}^{}s_{A}^{RR1}$		
**RR**	**1**	$w_{-}^{RR1}\;=\;1-{\;z}_{-}^{RR1}$	$w_{a}^{RR1\;}\;=\;w_{a}^{SS1\;}+s_{a}^{RR1}$	$w_{A}^{RR1\;}\;=\;w_{A}^{SS1\;}+s_{A}^{RR1}$		
**SS**	**2**	$w_{-}^{SS2}\;=\;1$			$w_{b}^{SS2}\;=\;1-\varphi_{b}^{SS2}$	$w_{B}^{SS2}\;=\;1-\varphi_{B}^{SS2}$
**RS (SR)**	**2**	$w_{-}^{RS2}\;=\;1-h_{-}^{2}{\;z}_{-}^{RR2}$			$w_{b}^{RS2\;}\;=\;w_{b}^{SS2\;}+h_{b}^{}s_{b}^{RR2}$	$w_{B}^{RS2\;}\;=\;w_{B}^{SS2\;}+h_{B}^{}s_{B}^{RR2}$
**RR**	**2**	$w_{-}^{RR2}\;=\;1-{\;z}_{-}^{RR2}$			$w_{b}^{RR2\;}\;=\;w_{b}^{SS2\;}+s_{b}^{RR2}$	$w_{B}^{RR2\;}\;=\;w_{B}^{SS2\;}+s_{B}^{RR2}$

Fitness costs of resistance may arise in mosquitoes which do not make contact with the insecticide. This reflects the possibility that the metabolic changes that enable IR may have deleterious effects on their normal metabolic function (e.g. [25, 26]). We therefore allow the option of fitness costs i.e. by setting z>0 in column “-”of Table 2 and these costs may exhibit different levels of dominance quantified by the associated ‘h’ parameter; fitness costs can be easily ignored (i.e. z = 0) if they are believed, or assumed, to be absent.

The third step is to define the initial frequency of the different genotypes in each sex. The simplest option is to define the starting frequency of resistance at each locus, and assume Hardy-Weinberg equilibrium (HWE) and linkage equilibrium (LE). The alternative is to define the genotype frequencies directly which is required if the assumptions of HWE and LE are violated; this may occur when one insecticide strategy is replaced by another as selection pressures for IR during the first strategy may have caused deviations from HWE and LE.

### Tracking the population genetics of IR genotypes

The basic assumptions made in this implementation, conventional in the population genetics literature (e.g. [22, 23]), are that individuals mate at random, have non-overlapping generations, and Mendelian inheritance.

The first step in the genetic model is to calculate the fitness of the diploid genotypes in each niche; this is achieved by multiplying the fitnesses of each single-locus genotype (summarised in Table 2) in the niche, to obtain the 2-locus genotypic fitnesses given in S1 Table of the Supporting Information. Our simulations will assume that the effects of insecticides in a mixture are multiplicative e.g. if the probability of surviving exposure to insecticide A alone is 0.3 and of surviving insecticide B alone is 0.2, then the probability of surviving expose to a mixture of A and B is 0.3*0.2 = 0.06. In other words, there is no synergy or antagonistic interactions between the insecticides. We do build flexibility into our methodology by scaling the fitnesses in niches where mosquitoes encounter both insecticides by a factor Λ_AB_, Λ_Ab_, Λ_aB_ or Λ_ab_ (S1 Table). If insecticides are synergistic then Λ<1, but if insecticides are antagonistic, for example because they share the same target site or share a similar mechanism of (cross)resistance, then Λ>1. This is a flexible approach, which we do not explore further here, but other strategies are also possible, for example if the insecticides are highly antagonistic then the fitness may be defined as the locus with the higher individual fitness factor, *w*.

The fitness of males and females of each 2-locus genotype are denoted by the upper case W to distinguish them from single locus fitnesses, *w*, on Table 2. Their values are their weighted average fitness across the nine different niches, the weighting reflecting the extent of their exposures (α) in each niche. For example, males carrying SS at both loci would have overall fitness as follows:

$$\begin{array}{l} W^{m,SS1SS2}=(\alpha_{-,-}^{m}W_{-,-}^{\;SS1\;SS2})+(\alpha_{a,-}^{m}W_{a,-}^{\;SS1\;SS2})+(\alpha_{A,-}^{m}W_{A,-}^{\;SS1\;SS2})+(\alpha_{b,-}^{m}W_{b,-}^{\;SS1\;SS2})+\\ (\alpha_{B,-}^{m}W_{B,-}^{\;SS1\;SS2})+(\alpha_{a,b}^{m}W_{a,b}^{\;SS1\;SS2})+(\alpha_{A,B}^{m}W_{A,B}^{\;SS1\;SS2}\;)+(\alpha_{A,b}^{m}W_{A,b}^{\;SS1\;SS2})+\\ \left(\alpha_{a,B}^{m}W_{a,B}^{\;SS1\;SS2}\right) \end{array}$$ (1)

Similarly, females carrying RS at locus 1 and RR at locus 2 would have overall fitnesses as follows:

$$\begin{array}{l} W^{f,\;RS1\;RR2}=(\alpha_{-,-}^{f}W_{-,-}^{\;RS1\;RR2})+(\alpha_{a,-}^{f}W_{a,-}^{\;RS1\;RR2})+(\alpha_{A,-}^{f}W_{A,-}^{\;RS1\;RR2})+(\alpha_{b,-}^{f}W_{b,-}^{\;RS1\;RR2})+\\ (\alpha_{B,-}^{f}W_{B,-}^{\;RS1\;RR2})+(\alpha_{a,b}^{f}W_{a,b}^{\;RS1\;RR2})+(\alpha_{A,B}^{f}W_{A,B}^{\;RS1\;RR2}\;)+(\alpha_{A,b}^{f}W_{A,b}^{\;RS1\;RR2})+\\ \left(\alpha_{a,B}^{f}W_{a,B}^{\;RS1\;RR2}\right) \end{array}$$ (2)

The remaining 16 fitness values (i.e. eight in each sex) are calculated in the same way. Note that we assume that the repulsion and coupling double-heterozygotes (see below) have the same fitness (S1 Table) although a difference could be encoded if required. We also assume males and females have the same fitnesses in each niche although, in principle, two versions of Table 2 could be constructed, one for males and one for females; this seemed like an over-elaboration in the current context. These fitness calculations describe selection due to insecticide exposure; they are independent of genotype frequency so need only be calculated once i.e. before the genetic model is run.

This is a diploid, 2-locus model. There are three genotypes at each locus (SS, SR, RR) making nine distinct 2-locus genotypes. However genotypes that are heterozygote at both loci are tracked as two types to allow for the fact that the two loci may be physically linked on the same chromosome and, if so, recombination may occur between the loci (S2 Table) i.e.

*Coupling heterozygotes* are where one chromosome is RR and the other SS

*Repulsion heterozygotes* are where one chromosome is RS and the other SR

Hence 10 diploid genotypes are tracked in the model. We need to track the relative frequencies of each genotype in each sex within the population. This is represented as F (the lower case ‘f’ is more conventional but we wish to distinguish frequency, F, from the female symbol, f). The first superscript to F indicates gender (male of female) while the second superscript indicates genotype at loci 1 and 2 so, for example, *F*^*m*, *SS*1*SR*2^ is the relative frequency, among males, of the genotype SS at locus 1 and SR at locus 2, and *F*^*f*, *RR*1*SR*2^ is the relative frequency, among females, of the genotype RR at locus 1 with SR at locus 2.

The first step is to obtain the genotype frequencies for the current mosquito generation. If the current generation is Generation 1 it takes the user-defined input values (which can, if desired, be genotypes that are not in HWE or LE), if not it takes the updated estimates of F’ produced in the previous generation of the simulation (see below).

The next step is to allow for mating among the survivors to produce the next mosquito generation. The most convenient way to do this is to calculate the relative frequencies of the four possible gametes from each sex (gamete outputs have to be calculated separately for each sex because survival of males and female genotypes may differ depending on their patterns of exposure to insecticides). The frequencies of gamete produced by each sex are denoted G where the subscript denotes the sex (m or f) of the gamete and the superscript the haplotype of the gamete, with the alleles at locus 1 given first and the allele at locus 2 given second. So, for example, $G_{m}^{SR}$ is the frequency of male gametes with the sensitive allele at locus 1 and a resistant allele at locus 2. The proportions of gametes produced by each diploid parental genotype are the product of three factors: the frequency of the parental genotype, its fitness in the face of insecticide deployment (cf Eqs 1 and 2), and the proportion of each type of gametes it produces. The latter is calculated according to the normal rules of meiosis with linkage and recombination. The calculations are summarised on S2 Table and gamete frequencies obtained by summing the production from each diploid genotype (i.e. summing within each columns in S2 Table). For example

$$\begin{array}{l} G_{m}^{SS}=\left[\left({\text{F}}^{\text{m,SS1SS2}}*{\text{W}}^{\text{m,SS1SS2}}*1\right)+\left({\text{F}}^{\text{m,SS1RS2}}*{\text{W}}^{\text{m,SS1RS2}}*0.5\right)+\ldots ‥+\left({\text{F}}^{\text{m,RR1RR2}}*{\text{W}}^{\text{m,RR1RR2}}*0\right)\right]/{\text{G}}_{\text{m}}\\ G_{m}^{RS}=\left[\left({\text{F}}^{\text{m,SS1SS2}}*{\text{W}}^{\text{m,SS1SS2}}*0\right)+\left({\text{F}}^{\text{m,SS1RS2}}*{\text{W}}^{\text{m,SS1RS2}}*0\right)+\ldots ‥+\left({\text{F}}^{\text{m,RR1RR2}}*{\text{W}}^{\text{m,RR1RR2}}*0\right)\right]/{\text{G}}_{\text{m}} \end{array}$$ (3)

And so on, where G_m_ is a normalisation coefficient that equals the sum of the numerators of the four male gamete production equations. A similar set of equations is required for females, again normalised by a coefficient G_f_.

Once these male and female gamete frequencies are calculated, random mating produces the diploid genotypes in the next generation which are conventionally denoted with a prime (F’). Both loci are assumed to be autosomal in the analyses presented here, although the model is structured to allow sex-linkage to be investigated, see later. For the autosomal case, female and male diploid genotype frequencies at the start of the next generation are identical. The details are in S3 Table and it is simply a case of summing within the rows of that Table. For example, genotypes SS1SS2 can only be formed by one gamete combination so

$$F^{\prime m,SS1SS2}=F^{\prime f,SS1SS2}=G_{f}^{SS}*G_{m}^{SS}$$ (4)

Genotype SS1SR2 can be formed by two gamete combination so

$$F^{\prime m,SS1SR2}=F^{\prime f,SS1SR2}=G_{f}^{SS}*G_{m}^{SR}+G_{f}^{SR}*G_{m}^{SS}$$ (5)

The other calculations can be re-derived from the details in S3 Table. Note that because the gamete frequencies are normalised within each sex, then the frequencies of diploid genotypes in each sex sum to unity. It is possible to adapt this approach to allow one of the loci to be sex-linked as described in the Supporting Information. These updated genotype frequencies, F’, are then used as the genotype frequencies in the next mosquito generation and the process is iterated over mosquito generations until a user-defined end-point is reached; typical endpoints are frequency of IR alleles exceeding a critical frequency.

We record, and present, two main types of genetic data. The frequency of IR alleles at each locus and the extent of linkage disequilibrium (LD) between the loci. The programme tracks both haploid (gametic) and diploid (genotypic) frequencies so LD can be measured directly from gametic frequencies, or inferred from genotypic frequencies. There are various measures of LD but here, for consistency, we report the metric used by Curtis [17] that uses gametic frequencies i.e.

LD=f(RR)−f(R1)f(R2) (6)

Where f(RR) is the frequency of gametes carrying the resistance alleles at both loci, and f(R1) and f(R2) are the frequencies of gametes carrying the resistance alleles at locus 1 and locus 2 respectively. Our model allows differential selection between the sexes so female and male gametes may have different allele and haplotype frequencies (this complication did not arise in Curtis as he assumed equal exposure of male and female mosquitoes to insecticide). In these circumstances we take the average of the male and female LD values because both contribute equal numbers of gametes to the next generation.

### The use of sub-models illustrated by the re-derivation of the results in the Curtis paper

**Fig 2:**
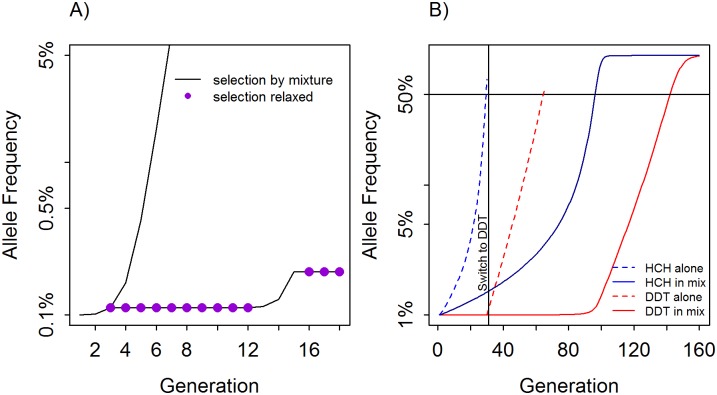
Using the methodology described in the main text to recreate Figs 1 and 2 from Curtis [17]. Panel (A) is our recreation of Curtis’s original Fig 1 in [17] which investigated a simple, idealised scenario: that insecticide is 100% effective against SS genotypes and has no effect against SR and RR genotypes. He tracked two simulations: one in which selection was continuous, and one where selection was “relaxed” selection (i.e. insecticide is not deployed) between generations 3 and 11 inclusive; the two lines are therefore superimposed up to generation 3. Panel (B) is our recreation of Fig 2 in [17] which simulated the deployment of the insecticides HCH and DDT and was calibrated using more realistic laboratory and field estimates of their effectiveness against the SS, SR and RR genotypes as described in the main text. Note that the switch to DDT in Panel (B) is only triggered the generation *after* the threshold of 50% is crossed (because the models use one-generation timesteps) so the level of resistance will inevitably 'overshoot' the threshold. These recreations of Curtis’s figures are indistinguishable from the originals, but we are unable to show the latter due to copyright regulations.

The methodology described above is designed to be flexible, comprehensive and, importantly, able to investigate specific scenarios by running sub-sets of the model. We demonstrate this application by re-deriving the results from previous analyses primarily by Curtis [17] who investigated IR in public health, and, as discussed later, other 2-locus models applied to agriculture; the intention is to show consistency between our simulations and to investigate how robust are the conclusion from that Curtis paper. All these publications made essentially the same assumptions i.e. 2-loci, resistance to each of two ‘insecticides’ encoded by different loci, equal exposure of sexes to the ‘insecticide’, and perfect deployment of ‘insecticides’ (we denote ‘insecticide’ in inverted commas because some studies (e.g. [11]) investigated plant-encoded insecticidal toxins rather than insecticides but the models are analogous). The process also serves as an illustration of how to investigate specific insecticide deployment patterns as subsets of our larger, nine niche, model. Figs 1 and 2 from Curtis [17] illustrated the use of insecticides alone or as mixtures. In this case the insecticide-free niche is, by definition “-, -”while the single insecticides are represented as niches “A, -“, “-, B” and the mixture as “A, B”. Other niches were omitted simply by setting exposure levels, α, to be zero for the five unrequired niches (Table 1). The fitness parameter in niches not required in the models (Table 2) are immaterial as they will never enter the calculation (they are scaled by their exposure value, i.e. multiplied by zero). The fitness parameters required to complete the calibration (Table 2) used by Curtis for his Figs 1 and 2 in [17] can be extracted from that paper as described in the SI. This information is sufficient for us to calibrate and run our model to replicate Fig 1 in [17]. His figure incorporates a period of relaxed selection when insecticide is not present, so the simulations must be stopped at generation 3, the genotype frequencies at time of stopping (generation 3) be retained, the exposure variables altered so that no insecticides are encountered, the model run until generation 12, the genotypes values retained and exposure variables changed back to reflect renewed insecticide deployment.

An influential assertion made in the Curtis paper is that mixtures may be counter-productive (made in the abstract and repeated on page 261) where Curtis refers to his Example (vi) on Table 1 in [17]. Unfortunately, Fig 1 in [17] only shows the spread of resistance using a mixture and does not quantify the extent to which it is inferior to sequential use. However the calibration used to replicate Fig 1 in [17] allows us to extend the one-generation argument presented in Table 1(vi) in [17] over the numerous generations required to simulate the spread of resistance (see later discussion of our Table 3). Figure 2 in [17] examined a more realistic set of genetic parameters, calibrated against field data, for two insecticides DDT and HCH where resistant and sensitive homozygotes survive DDT exposure with fitnesses of 50% and 27% respectively (caption to his Fig 2); the calibration can be extracted from Curtis data into the form required by our model as described in our Supporting Information.

**Table 3:** Investigation of the two insecticide deployment scenarios presented by Curtis using arbitrary data on insecticide resistance. Curtis argued that mixtures may sometimes be counterproductive compared to sequential use of the insecticides so, following Curtis, we defined time to resistance as the time, in mosquito generations, until resistance to both insecticides exceeds 50%. The Curtis study may also be interpreted (see Discussion in main text) as postulating that resistance to the insecticide with a lower starting frequency of resistance may spread faster if deployed in a mixture compared to being deployed alone; we therefore calculated the time, in mosquito generations, for resistance to reach 50% for that insecticide under the two policies. Note that the starting frequency of resistance at the locus with a higher frequency is immaterial when the insecticide with lower starting frequency is deployed alone. We have assumed that the insecticide with lower levels of resistance would be a “newer” insecticide replacing an older insecticide where resistance had already reached relatively high frequencies; we therefore assume the “new” insecticide is the one deployed second in a sequential deployment. The idealised example presented in Table 1, example (vi) in [17], assumed both resistance alleles are fully dominant and have different starting frequencies; Note that the fourth row, where frequency of starting resistance at loci A and B are 0.01 and 0.001 respectively corresponds to the specific case given by Curtis (Table 1, example (iv) in [17]) which is recreated as described in the main text. Locus B encodes the rarer allele so the time for it to reach 50% determines the lifespan of the deployment so these are the parameters presented in the results column.

Locus A starting Frequency	Locus B starting Frequency	time to resistance Ratio Mix/Seq	Time to 50% (B) alone	Time to 50% (B) in Mix
0.01	10^-2	**6/7 = 0.86**	**3**	**7**
0.1		**5/6 = 0.83**	**3**	**6**
0.3		**4/5 = 0.80**	**3**	**4**
0.01	10^-3	**8/8 = 1**	**4**	**8**
0.1		**6/7 = 0.86**	**4**	**6**
0.3		**6/6 = 1**	**4**	**6**
0.01	10^-4	**9/9 = 1**	**5**	**9**
0.1		**8/8 = 1**	**5**	**8**
0.3		**7/7 = 1**	**5**	**7**

### Sensitivity analysis of the model

Curtis used single examples to illustrate and develop his arguments. Substantial improvements in computer power and simulation software in the subsequent 30+ years enable more sophisticated investigations to be undertaken. We illustrate this approach by performing a sensitivity analysis around the results shown on Fig 2 in [17] in which Curtis shows that mixtures perform better than sequential use for a single parameter set based on field and lab data. We do this as follows. As in Curtis, (1) Only two niches are present in any run i.e. no-insecticide plus insecticide-present (either as a single insecticide or as a mixture). (2) The simulations are started in Hardy-Weinberg equilibrium with linkage equilibrium between loci (3) Natural selection coefficients (z in Table 2) are set to zero; consequently the values of their dominance coefficients are immaterial (so z = h = 0 in all equations in the “-, -” column of Table 2).

We then ran 10,000 simulations varying the remaining parameters independently within the value ranges in Table 4. These parameters can be briefly summarised as follows.

**Table 4:** Variables used in the main text, Figures and Tables. Their definitions, the corresponding symbols used in Equations, and their parameter distributions used for sensitivity analysis.

Variable name	Definition	Symbol	Parameter distribution (uniform unless stated)
start_freq_allele1 start_freq_allele2	Starting frequency of resistant allele at locus 1 and 2.	n/a	0.0001–0.1 (log uniform)
exposure	Proportion of female mosquitos exposed to insecticide.	Α (Table 1)	0.1–0.9
male_exposure_prop	Male exposure to insecticide as proportion of female exposure.	n/a	0–1
effectiveness_ins1 effectiveness_ins2	Proportion of SS mosquitoes killed after contact with insecticide 1 or 2	$\varphi_{A}^{SS1},\;\varphi_{B}^{SS1}$ (Table 2)	0.3–1.0
dominance_allele1 dominance_allele2	Dominance of resistance at locus 1 and 2	h_A_, h_B_ (Table 2)	0–1
rr_restoration_ins1 rr_restoration_ins2	The ability of the RR genotype to restore the fitness that has been reduced by the insecticide	n/a	0.2–1
correct_mix_deploy	Percentage of insecticide treatment that is deployed correctly.	n/a	0.5–1

- Starting frequencies of resistance: we use a log-uniform distribution to allow sampling of low values i.e. 10^-x^ where x is uniformly sampled.
- Exposure to insecticide: the proportion of female mosquitoes that encounter insecticides.
- A “male exposure” parameter which defines the proportion of males exposed to insecticide as a proportion of the female exposure; it is assumed to be the same for each insecticide and niche. Assuming, for example. that female exposure is as follows: unexposed = 0.2; AB = 0.56; A = 0.12; B = 0.12. If "male exposure" is 0.6, then male exposure to niches ‘AB’, ‘A’, and ‘B’ are 0.56*0.6 = 0.336, 0.12*0.6 = 0.072 and 0.12*0.6 = 0.072 respectively and the proportion unexposed rises to 0.2 + (0.8*0.40) = 0.52.
- Effectiveness of insecticide 1 & 2 is defined as the proportion of SS mosquitoes killed after contact with insecticide (i.e. the four *φ* values in Table 2). The fitness of SS genotypes in presence of the insecticide to which it encodes resistance are calculated as 1 minus these effectiveness inputs.
- Dominance coefficients for allele 1 & 2: the “h” coefficients in Table 2.
- Two “rr restoration coefficients” for insecticides 1& 2. These coefficients are used to generate the selective advantage of the RR genotypes (i.e. the six *s*^*RR*^ coefficients in Table 2). It is not possible to define the selection coefficients *s*^*RR*^ directly, because high values could enable the fitness of RR genotypes to exceed 1. Values >1 are not acceptable because the SS genotype in the absence of insecticide is assumed to have the maximum possible fitness in the population and is assigned the reference fitness value of 1 (see Table 2). We avoid this possibility by noting that the maximum value of selection coefficient that can occur and the RR fitness still be ≤1 is 1 − *ω*^*SS*^ and Table 2 shows that 1 − *ω*^*SS*^ = (1 − *φ*^*SS*^) = *φ*. The rr_restoration parameter quantifies the ability of the RR genotype to overcome the effect of the insecticide and restore mosquito fitness in the presence of insecticide towards it maximum value of 1.0 i.e.the selection coefficients of the RR genotypes are calculated as the rr_restoration_coefficient times *φ*. For example a rr_restoration parameter with value 1.0 means the RR genotype is completely unaffected by the insecticide (the RR genotypes in the presence of the insecticide have the same fitness as SS genotypes in the absence of insecticide i.e. 1.0) while a rr_restoration parameter with value of 0 means the ‘resistance’ mutation is completely ineffective i.e. that the RR genotype has the same fitness as the SS genotypes (we ignore the possibility of over-dominance).
- A “correct mixture deployment” parameter is incorporated in the simulations investigating the use of mixtures. Correct deployment is often not achieved for a variety of operational reasons and exposure to insecticides may not be as anticipated. In particular, mixtures may be mandated but not achieved (e.g. due to stock-outs) such that they are replaced by the single insecticide that is available. In our simulation the incorrectly exposed mosquitoes are equally divided into exposure to each of the single insecticides. e.g. if exposure is 0.8 and "correct mixture deployment" is 0.7 then 0.8 x 0.7 = 0.56 are exposed to the mixture, while [0.8x0.3]/2 = 0.12 are exposed to insecticide 1 and 0.12 to insecticide 2. In reality, poor deployment will result in exposure to different concentrations of insecticides in both mixture and sequential applications and our model could be used to investigate these implications further.

It is necessary to identify thresholds or 'critical points' in insecticide deployment strategies to define the time at which an insecticide or mixture is deemed of no further value (and, in the case of a sequence strategy, be replaced by the next insecticide). This threshold is defined here in terms of frequency of resistance alleles. Plausible values would be 10%, 25%, 50%; here we follow Curtis and use 50% (c.f. his Fig 2). This has been used in other modelling studies on IR (e.g. [19] and resistance in other organisms (e.g. [27]), but see Gould [11] for an example of thresholds based on mosquito viability. The timescale until these thresholds are reached are measured in units of mosquito generations. The number of mosquito generations per year depends on the vector species and their local environment/ecology but a figure of one generation per month, or 12 generations per year seems reasonable and enables the timescale to be easily converted from generations into years. Three deployment policies are investigated as follows.

#### Sequential deployment

One insecticide is deployed until its resistance frequency reaches the 50% threshold, at which point it is replaced by the second insecticide until its resistance allele frequency also reaches the threshold. Thus the time to resistance of the sequential strategy is defined as the number of generations for the two insecticide resistance alleles frequencies to exceed 50%.

#### Mixture deployment

The two insecticides are deployed at the same time as a mixture and the time to resistance is defined as the number of generations until both resistance allele frequencies exceed 50%.

#### Adaptive mixture deployment

The mixture is deployed until one resistance allele reaches the 50% threshold, then, to save costs, the insecticide to which resistance has not yet reached the critical point is deployed alone. Time to resistance is defined as the number of generations until both resistance allele frequencies exceed 50%. This option is intermediate between mixture and sequential deployment and its time to resistance can be estimated from the data generated during these two deployment types. The time until one allele frequency exceeds the threshold is obtained from the data recorded in the mixture deployment. The time for the replacement single-insecticide to reach 50% is obtained from the data produced in sequential use using the starting frequency at the time the mixture is replaced by the single insecticide; this ignores differences in HWE and LD that may have arisen during the selective processes but these take just a few generations to return to background levels in the absence of selection.

The results obtained from the sensitivity analysis were analysed using partial rank correlation coefficients (PRCC) and by generating classification trees (as in a previous study of insecticide use and strategy decisions; see Fig 4 of Barbosa et al [24] and associated discussion). PRCC is analogous to standard (Pearson) correlation but takes account of other parameters in the dataset. It is interpreted in the standard manner: a value of zero indicates no relationship between variable and outcome, values close to +1 or -1 indicate a variable with a high impact on the outcome, and the signs of the coefficients indicate the direction of the relationship. The variables given in Table 4 were included in a classification tree as was the ratio of the higher to lower starting frequency because Curtis had speculated that this ratio (i.e. the discrepancy in initial starting frequencies) could be important. To avoid overfitting, the number of levels in the classification trees was restricted to the minimum where the relative error plus the standard error was less than the cross-validation error. We generated two classification trees. One where mixtures were deemed superior if their time to resistance was longer than the time to resistance under sequential use, and another where mixtures were only deemed superior if the time to resistance was >20% longer than for sequential use.

Runs where the resistance threshold of 50% was not reached within 500 generations were not included in the sensitivity analysis. The reasoning behind this decision is that, assuming a mosquito generation lasts one month, then 500 generations represents a timeframe of around 42 years. Realistic strategy considerations act over a much shorter timeframe and we are only interested in decisions made in this timeframe, hence our focus on simulations where resistance arises in less than 42 years.

The model is implemented in the statistical environment R [28] and the sensitivity analysis uses the packages “sensitivity” and “rpart”. The code is available at https://github.com/AndySouth/resistance and on request. A user interface demonstrating the model is available at https://andysouth.shinyapps.io/shinyFig2Curtis/. It shows how the model can generate Fig 2 in [17] and allows the user to change parameter values to examine the consequences of doing so.

## Results

We successfully recreated Figs 1 and 2 in [17] as shown on Fig 2 of this paper. These figures presented simulations based on single sets of parameter values so we extended the number of parameter sets upon which they were based. We first investigated the assertion of Curtis (which he did not quantify) that mixtures could be counterproductive if deploying a new insecticide when resistance to both insecticides was dominant, and the frequency of resistance to the ‘new’ insecticide was lower that frequency of resistance to the ‘old’. This assertion appears to be true over a range of resistance starting frequencies using the simple example he presented in his Table 1, example (iv). The differences were relatively small with sequential use lasting for at most a single mosquito generation longer (the”time to resistance ratio Mix/seq” column in Table 3). Resistance spreads extremely rapidly under his assumptions (complete survival of RS and RR genotypes) and the insecticides became ineffective within a few generations of either strategy. Consequently, the differences in time to resistance between mixtures and sequential use were relatively small so these simulations were rather insensitive metrics for comparing the relative merits of mixtures and sequential use. It is debatable if this level of insecticide exposure and intense selection is likely to occur in real deployments (although we note extremely high coverages of ITNs have been reported). We then relaxed this assumption of complete dominance at both loci and explored the relative merits of mixture and sequential deployment using Curtis’s HCH and DDT example (i.e. the calibrations used in Fig 2 in [17]). The results are given on Table 5. The results consistently showed that deploying insecticides as mixtures increases their time to resistance compared to sequential deployment, with time to resistance being ~2.5 fold to ~4 fold longer when deploying insecticides as mixtures.

**Table 5:** Investigation of the two insecticide deployment scenarios presented by Curtis using field data on insecticide resistance. As for Table 3 but investigating the Curtis example based on field and laboratory data on gamma-hexachlorocyclohexane (HCH) and dichlorodiphenyltrichloroethane (DDT) deployment (Fig 2 in [17]). HCH resistance is the rarer of the resistance alleles when deployment is initiated so is invariably the rate-limiting factor in the dynamics and the time for that allele to reach 50% in a mixture determines the lifespan of the mixture i.e. the time for both alleles to exceed 50%. Similarly, when HCH is deployed in a sequence, resistance to DDT spreads very rapidly from its relatively high frequency so it is the time taken for HCH resistance to reach 50% that largely determines the lifespan of the sequential deployment.

DDT(R) starting Frequency	HCH(R) starting Frequency	time to resistance Ratio Mix/Seq	Time to 50% HCH alone	Time to 50% HCH in Mix
0.1	10^-2	**110/40 = 2.75**	**20**	**90**
0.3		**90/35 = 2.57**	**20**	**82**
0.5		**78/30 = 2.60**	**20**	**78**
0.1	10^-3	**580/150 = 3.87**	**150**	**550**
0.3		**520/150 = 3.47**	**150**	**510**
0.5		**480/150 = 3.20**	**150**	**480**
0.1	10^-4	**1700/500 = 3.40**	**500**	**1700**
0.3		**1500/500 = 3.00**	**500**	**1500**
0.5		**1380/500 = 2.76**	**500**	**1380**

**Fig 3:**
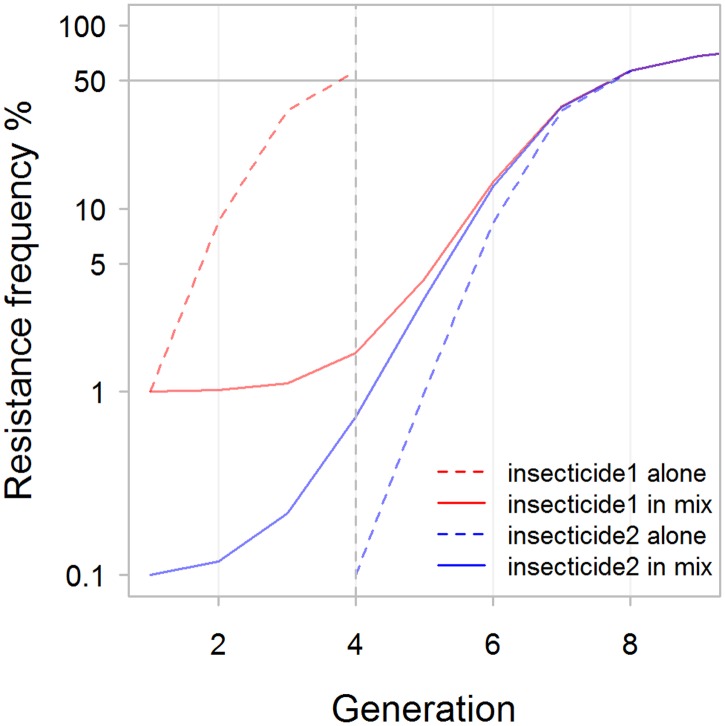
Recreating example vi of Curtis as given in his Table 1 in [17]. Curtis reported calculations and results for a single generation of selection under a range of assumptions in his Table 1 (note: this table is absent from many current electronic reprints). He made particular use of his example (iv) to develop influential arguments about the (dis)advantages of using an insecticide mixture when the initial frequencies of resistance are unequal. We therefore extended this example to plot the subsequent, multi-generational dynamics of resistance spread assuming, as he did, that the first insecticide deployed in a sequence become non-beneficial, and is switched when alleles encoding resistance to the insecticide reach 50%. Resistance to both insecticides exceeded 50% by generation 8, irrespective of whether they were deployed in sequence or as a mixture.

We next investigated the statement by Curtis (page 261), that “the use of a mixture where the initial gene frequencies are unequal leads to more rapid increase in the frequency of the rarer of the genes”. The context of this statement has been widely interpreted as an assertion that resistance to the insecticide with lower levels of resistance will spread faster when that insecticide is used in a mixture compared to if it is used alone (although we could not reconcile this interpretation of its context with the data; see later discussion). Curtis’s example was based on his simple example (his Table 1, example (vi)) and on selection over a single-generation. We therefore ran this example over a number of generations to allow for linkage disequilibrium to build up. The results are shown on Fig 3 and are inconsistent with the above interpretation i.e. our results show that resistance to an insecticide spreads faster when the insecticide is deployed alone than when deployed in a mixture (although in this example the overall sequential and mixture operational lifespans are equal). We then varied the relative frequencies of resistance and again found resistance always spreads faster when the insecticide with lower frequency of resistance is deployed alone, than if it is deployed in a mixture (last two columns of Table 3). We then performed the same calculations for Curtis’s HCH/DDT example and again found resistance always spreads faster if deployed alone than in a mixture (last two columns of Table 5).

After recovering the results of Curtis, we moved on to a sensitivity analysis of strategy choice in our parameter space. Twenty four percent of sensitivity runs did not reach the resistance threshold within 500 generations and were excluded from further analyses as the timescale is so long that both strategies can be regarded as operationally equivalent. These runs were characterised by low values of starting frequencies, exposure and dominance (as would be expected from the model equations). We could, of course, vary these input ranges so that >95% of runs reach the threshold in less than 500 generation, however we did not wish to restrict our coverage of parameter-space. In particular, we wanted to include low levels of starting frequency because, under these circumstances most resistance alleles will occur in heterozygotes and dominance becomes important under these circumstances.

**Fig 4:**
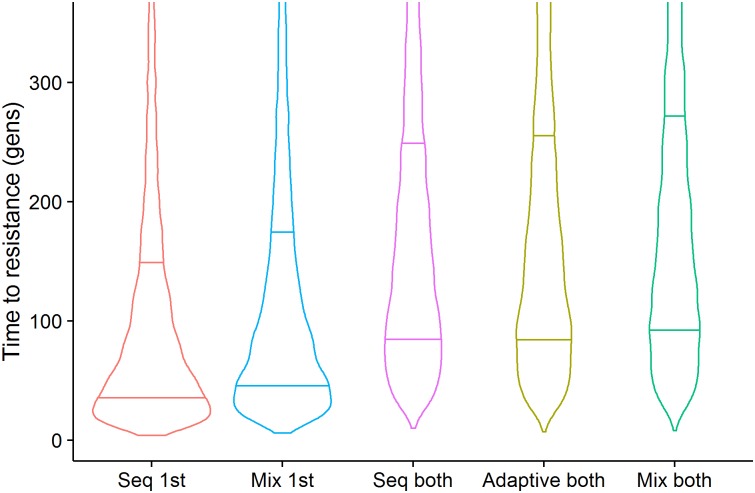
A Violin plot of the times, in mosquito generations, for resistance allele frequency to reach 50%. The width of the plot indicates density of points and the horizontal bars the 25% and 75% quartiles. The results were obtained from the sensitivity analysis described on Table 4. “Seq 1st” is the distributions of times until resistance to the first insecticide deployed in the sequence reaches 50%. This is equivalent to the times that would occur if insecticides are used alone. “Mix 1st” is the time until resistance reaches 50% against either insecticide deployed in a mixture. “Seq both” is the time until resistance has exceeded 50% to both insecticides that were deployed in sequence. “Adaptive both” shows the time until resistance is reached for both insecticides in a mixture when the first to reach resistance has been adaptively withdrawn (because there is widespread resistance to it). “Mix both” is the time until resistance alleles frequency exceeds 50% for both insecticides in a non-adaptive mixture.

**Fig 5:**
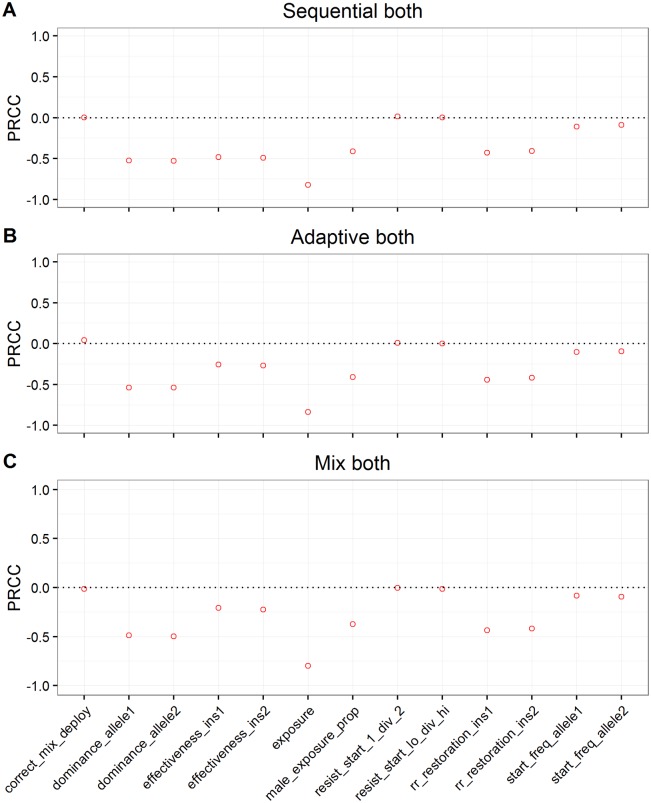
The partial rank correlations (PRCC) between the parameter values of the sensitivity analysis (Table 4) and times to resistance under each deployment strategy. A value of zero (represented by the horizontal dotted line) indicates no relationship while increasing distance from zero, up to the maximum value of 1 or -1, indicates parameters with increasing impact on times to resistance. The variable names on the X axis are as described and defined on Table 4, except for resist_start_1_div_2 which is the starting frequency of the resistance allele at locus 1 divided by the starting frequency of the resistance allele at locus 2. Similarly, resist_start_hi_div_lo is the higher of the two starting frequencies of resistance divided by the lower frequency. These ratios were included because Curtis [17] speculated that they may be important determinants of strategy choice. The output variables analysed were times for both resistance allele frequencies to exceed 50% for three deployment strategies summarised in Fig 4 i.e. Panel (A) is time under a policy of sequential deployment, Panel (B) is time when deployed as an adaptive mixture, and Panel (C) is time when the insecticides are always deployed as mixture.

**Fig 6:**
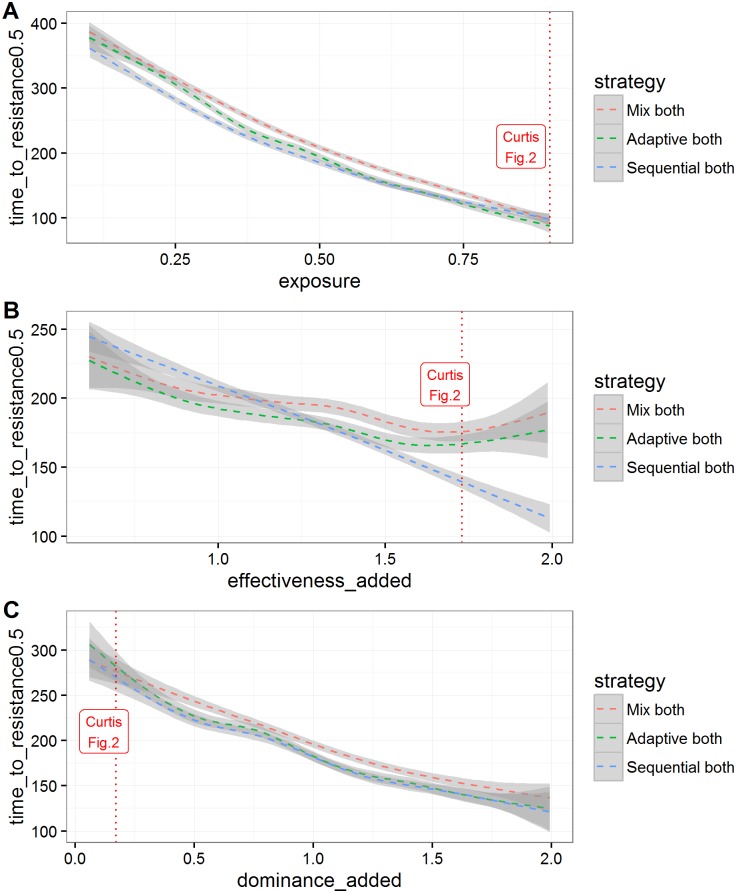
Examples of the relationship between time in mosquito generations until resistance allele frequency reaches 50% and three key variables (as identified by PRCC) in the sensitivity analysis. The X axis parameters are as defined in Table 4 which also gives their distributions in the sensitivity analysis. The lines are smoothed conditional means whose surrounding grey areas are their 95% confidence intervals. The red dotted lines indicate the parameter values used by Curtis in Fig 2 in [17]. Panel (A) is time to resistance as a function of mosquito exposure to insecticide. Panel (B) is time to resistance as a function of the effectiveness of the insecticides against the SS genotypes, the effectiveness of each insecticide being summed to construct the X-axis variable. Panel (C) is time to resistance as a function of the dominance of the resistance alleles, the dominance values being summed to construct the X-axis variable.

In those runs where resistance allele frequencies did exceed 50% in less than 500 generations, 56% favoured sequential use and 44% favoured mixtures. The distributions of times to resistance in the sensitivity analysis differed for each deployment strategy but with considerable overlap between them (Fig 4). Mean time to resistance was shortest when insecticides were used alone (plotted as “Seq 1^st^” on Fig 4) and longest for the mixture strategy. In between these extremes mean time to resistance was slightly shorter for the adaptive mixture strategy, as would be expected because removing the “failing” insecticide removes any lingering small-scale protection it may have afforded in the mixture. The relative influence of the parameters on times to resistance was similar across strategies as shown by their partial rank correlation coefficients (PRCC) on Fig 5. Insecticide exposure had the greatest influence in all strategies with their PRCC coefficients of absolute magnitude greater than 0.75. Insecticide effectiveness (which will later be shown to be important for determining whether sequential use or mixtures are optimal) has a negative impact on times to resistance in sequential use (PRCC -0.5), but has less of an impact in mixtures. Examples of the relationship between these key parameters and times to resistance are shown on Fig 6. Exposure and dominance have a consistent effect on each strategy whereas, in contrast, effectiveness has less of a negative effect on the mixture strategies than the sequential.

**Fig 7:**
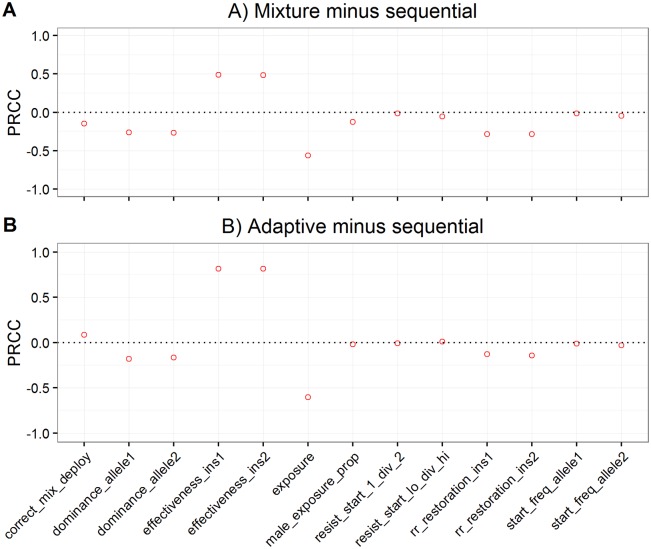
The partial rank correlations (PRRC) between the parameters of the sensitivity analysis (Table 4) and strategy choice. The effect of strategy choice was calculated as follows: For each of the 10,000 parameter combination in the sensitivity analysis, the time to 50% resistance was recorded for sequential, mixture and adaptive mixtures and used to calculate (A) time to resistance in mixture minus that under sequential deployment. (B) time to resistance in adaptive mixture minus that under sequential. X axis parameters are as defined in Table 4 except from resist_start_1_div_2 and resist_start_hi_div_lo which are defined in the caption to Fig 5.

The analyses shown so far (i.e. Figs 4 to 6) all investigated deployment strategies separately. We now focus on direct comparisons of the difference in times to resistance between strategies within each parameter set. Fig 7 shows the PRCC between these differences and the input values. For the difference in time to resistance between the standard mixture and sequential strategies the most influential parameters (i.e. with coefficients greater than 0.5) were insecticide exposure and the effectiveness of the insecticides (Fig 7A). Similar results were obtained for the adaptive mixture strategy compared to Sequential use (Fig 7B). Note the key difference of these PRCC results compared with the PRCC results shown in Fig 5 i.e. that insecticide effectiveness now has a large coefficient and hence a large influence on determining which deployment strategy is optimal under any given set of parameters. Curtis putatively identified two factors that differentially affect whether mixtures or sequential use is favoured: unequal starting frequencies and dominance. Interestingly, neither had a particularly large impact in our sensitivity analysis where effectiveness of the insecticides and exposure were the main differentiating factors.

**Fig 8:**
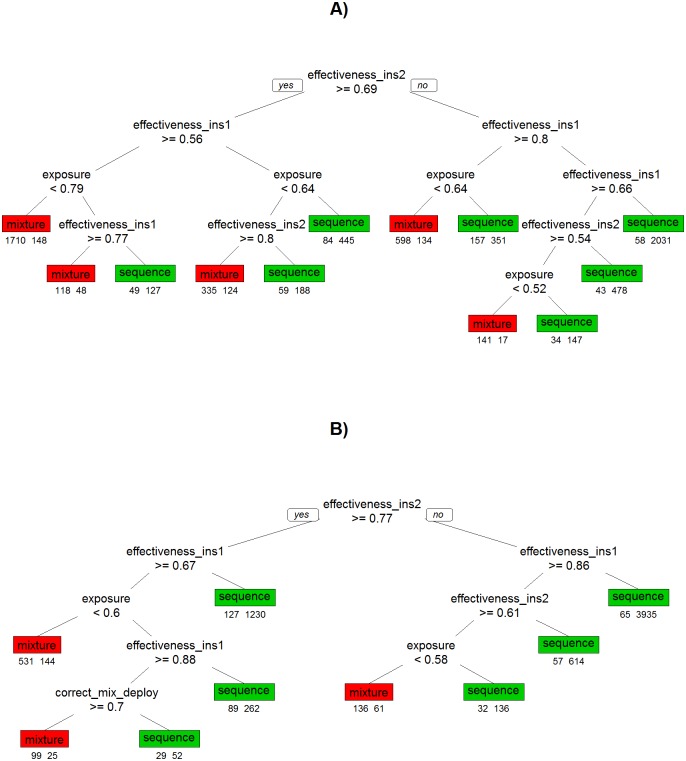
Classification tree from the sensitivity analysis showing under what circumstances sequential deployment is superior to using mixtures, or *vice versa*. The decision nodes indicate whether to move to the left (if the statement is true), or to the right (if the statement is false). The final classification boxes are shaded red if mixtures are the most favoured outcome having followed that decision path, and are shaded green if sequential use if favoured. The numbers below each classification box are the number of simulations in that box favouring Mixture on the left and Sequence on the right. (A) Mixtures are deemed superior to sequential deployment if their time to resistance is longer than Sequential deployment (B) Mixtures are only deemed superior to sequential deployment if their time to resistance is at least 20% longer than Sequential. Parameter names are as defined on Table 4.

**Fig 9:**
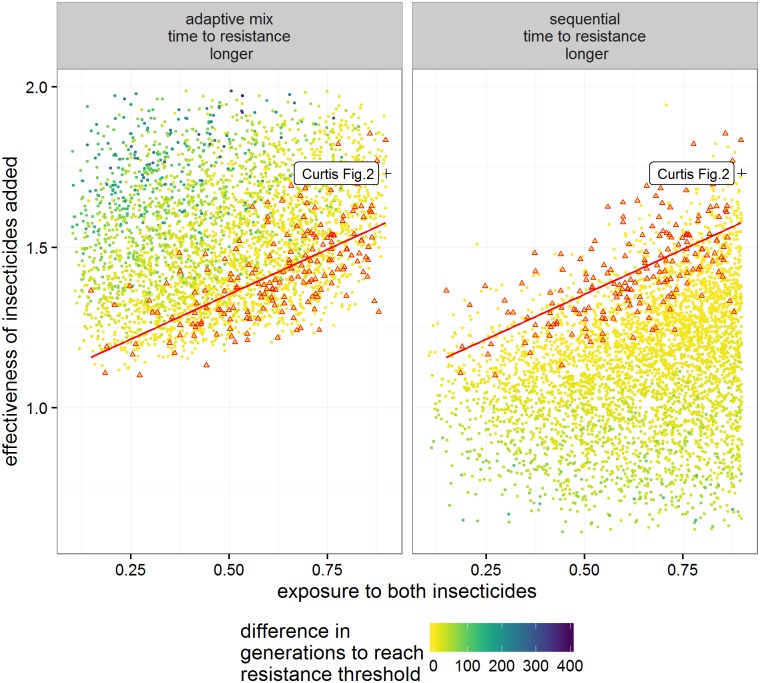
Using insecticide effectiveness and exposure to determine whether time to resistance to the insecticides is longer using Adaptive mixture or Sequential strategies. Our analyses suggested that insecticide effectiveness and levels of insecticide exposure were key determinants of whether mixtures outperformed sequential deployment, or *vice versa*. We investigate how well they combine to predict optimal strategy by constructing this figure based on two axes: (i) “Effectiveness”, defined as the sum of effectivenesses of insecticide 1 and 2 against the SS genotypes(see Table 4 for details), and (ii) “Exposure”, defined as the proportion of female mosquitoes that encounter the insecticide. The difference in times until resistance under the two strategies are indicted by colour coding as shown on the Figure key. The black cross indicates the values used by Curtis in his Fig 2 in [17]. Red triangles are those runs in which time to resistance is identical for the two strategies (and are repeated in both panels). The red line is a linear model through these same points, and is described by Eq 7 in the main text.

Classification trees based on the sensitivity analysis are shown on Fig 8. Their results are similar to those obtained by PRCC analyses (Fig 7) i.e. effectiveness of the insecticides in killing the sensitive genotypes was the primary variable driving decisions. The advantage of classification trees over PRCC is their utility as a decision support tool which is discussed below. So far, we have looked at the absolute difference in time to resistance between the sequential and mixture strategies. Mixtures would involve using more insecticide and therefore to be favoured in a cost-benefit analysis may need to demonstrate greater long-term benefits. We can take this into account by making a requirement that time to resistance must be >20% longer in the mixture and this is shown on Fig 8B. Finally, having identified insecticide effectiveness and exposure as the key inputs identified in the classification trees and PRCC analysis, we attempted informally to construct a simple metric that would predict whether sequential deployment or use of mixtures would be favoured. We summed the effectivenesses to insecticide 1 and 2 and used this metric with exposure to generate a plot showing how these variables affect the time to resistance (Fig 9). This informal approach resulted in a good separation between those runs in which time to resistance was longer in each of the insecticide use strategies. The mixture strategy is favoured by lower values of exposure and higher values of effectiveness. Within the parameter space investigated mixture strategies can provide greater benefits with time to resistance being more than 200 generations longer than in sequential strategy (upper left of Fig 9). In contrast for the parameter space where times to resistance are longer in sequential strategies (lower right of Fig 9) the differences are generally less than around 100 generations. We identified (and highlight on the Figure) those runs in which the time to resistance was reached at the same time in the two strategies, and fitted a linear model to them. Such a linear model could be used to predict when time to resistance might be expected to be longer using mixture or sequential strategies. The empirically-derived putative decision line $\hat{D}$ shown on Fig 9 is given by

$$\hat{D}=1.07+0.56*\text{exposure}$$ (7)

**Fig 10:**
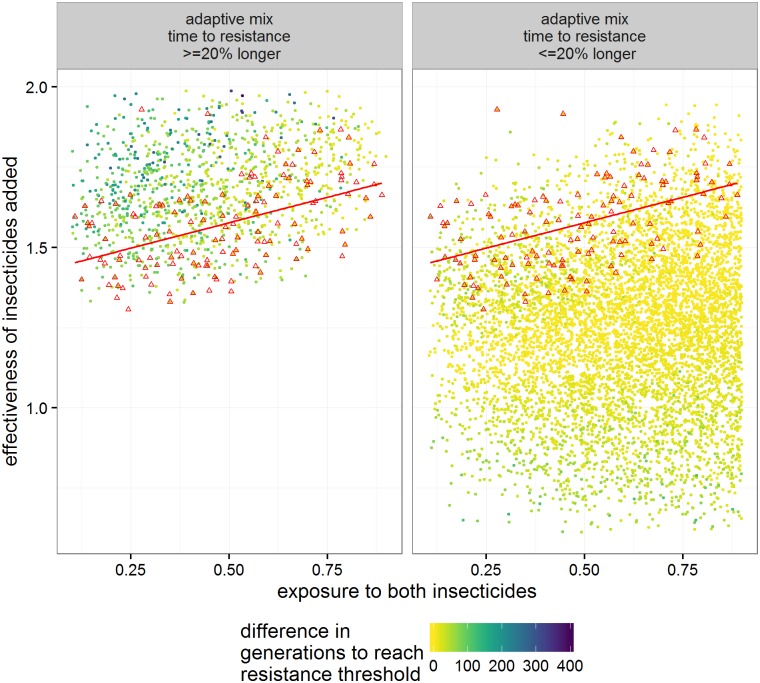
As for Fig 9 except that the deployment decision is based on whether an Adaptive mixture can increase the time to resistance by at least 20% compared to Sequential. The red triangles now indicate run where time to resistance in Adaptive mixtures are between 19 and 21% longer than in a Sequential strategy. The gradient of the red line is given in Eq 8 in the main text.

Prediction of best strategy based on this line results in a true positive rate of 0.82 and a true negative rate of 0.95 and the consequences of being wrong are likely to be small: the colour coding on Fig 9 indicating that differences in time to resistance in the misclassified cases are likely to be in the region of <20 generations. Furthermore, this is over all parameter space so narrowing parameters space down, as may occur when specific deployment locations are known, may well decrease the misclassification rate. We also attempted this for the situation where time to resistance of mixtures must be >20% longer to be favoured over sequential use; the data with the empirically-derived putative decision line $\hat{D}$ are shown on Fig 10 where

$$\hat{D}=1.42+0.32*\text{exposure}$$ (8)

The number of runs in which the mixture strategy is favoured are reduced, remaining in the upper left of the plot at low values of exposure and high values of effectiveness. The classification has a true positive rate of 0.78 and a true negative rate of 0.93.

**Fig 11:**
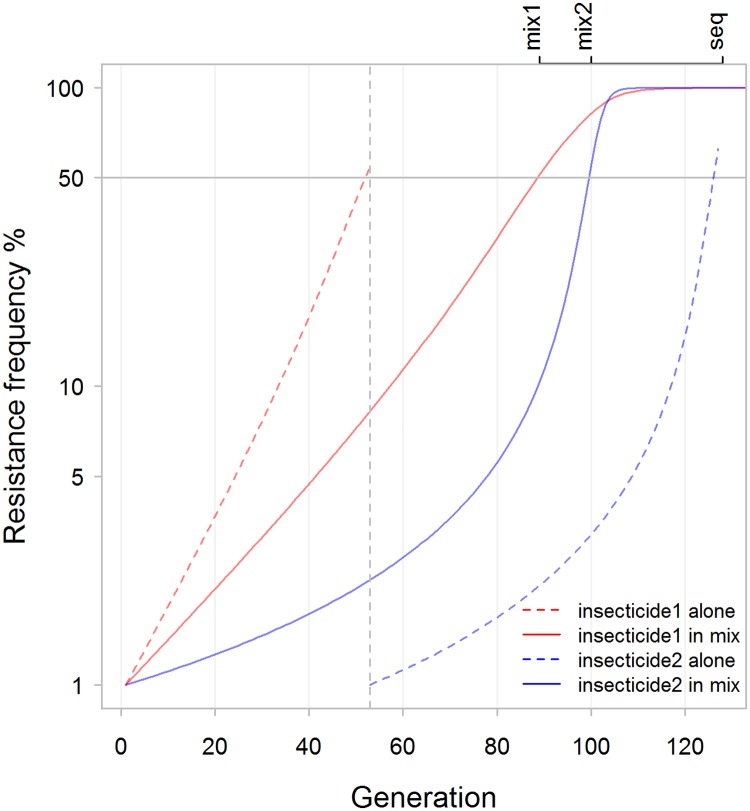
An example of a parameter combination in which resistance develops more slowly when insecticides are used in sequence relative to when they are used in a mixture. The labels on the upper margin of the plot mark where the resistance threshold is reached for the second insecticide in a sequence ('seq') and the first and second insecticides in a mixture ('mix1' and 'mix2'). Note that resistance spreads faster for both insecticides when they are deployed on their own (dashed lines) than when they are deployed in a mixture (solid lines). The reason sequential use is favoured is because of the time delay that occurs before insecticide #2 is deployed in the sequence. In this example, the frequency of resistance alleles to both insecticides in the mixture exceeded 50% at generation 100. Resistance allele frequency to insecticide 1 deployed alone required 53 generations to exceed 50% (at which point it was replaced by insecticide 2 as indicated by the vertical dotted line). It then took 75 generations for resistance allele frequency to exceed 50% for insecticide 2 deployed alone. The total of these timescales, i.e. 53 + 75 = 128 generations, is greater than the 100 generations for resistance to evolve to the mixture, indicating that sequential deployment is favoured in this simulation. [parameter combinations are as used for Curtis in his Fig 2 in [17] (our Fig 2B) with the exception that each effectiveness was reduced by 0.2 to 0.53 for insecticide1 and to 0.8 for insecticide2]

It is intuitively unlikely that resistance to an insecticide would spread slower when used alone than when used as a mixture (because the second insecticide would be expected to have at least some protective effect and impact in slowing resistance to the first insecticide). We therefore interrogated our data and confirmed that this was indeed the case, again using time for resistance to reach 50% as the criterion. In every case, irrespective of whether mixtures or sequential use was eventually favoured, each insecticide lasted longer when deployed as part of a mixture than when deployed on its own. An obvious question is how can sequential use sometimes be favoured even though resistance to individual insecticides always spreads faster if the insecticide is deployed on its own? The answer seems to be that the time delay until the second insecticide is deployed is sometimes sufficiently long to offset the greater rate of spread of resistance to each insecticide deployed individually. As a hypothetical example, two drugs may last 3 and 4 years each when deployed alone but 5 years when deployed as a mixture: resistance spreads slower to each in a mixture but sequential use lasts longer i.e. 3 + 4 = 7 years compared to 5 years for a mixture. A specific example of this is shown on Fig 11 which plots the dynamics of a simulation where sequential use is favoured.

## Discussion

The main purpose of this manuscript is to develop, implement and demonstrate a flexible, two-locus model of insecticide resistance that can incorporate factors such a differential exposure of females and males to insecticide-based interventions, the impact of poor adherence to deployment recommendations, and other factors such as physical linkages of the two loci on an autosome, or the presence of one locus on the X chromosome. It is explicitly aimed at public health deployments. A parallel literature has developed in agricultural insecticides (op. cit. i.e. [11–16]) but differs somewhat in that the problems and insect species addressed are different (which make it less relevant to public-health audience) and because agricultural use is relatively well funded and often occurs in a well-developed infrastructure that makes deployment relatively precise. For example, strict seasonal-rotations of different insecticides can be proposed and enforced.

The secondary objective was to apply the methodology to re-examine the highly influential paper by Curtis [17]. Notably, the abstract of this paper states

“*The use of a mixture appears advantageous provided that resistance is not fully dominant, but, if it is, linkage disequilibrium builds up rapidly and nullifies the advantage of the mixture*”.

Later, on page 261, Curtis states that

“*Example vi shows the, at first sight unexpected, point that the use of a mixture where the initial gene frequencies are unequal leads to more rapid increase in the frequency of the rarer of the genes. This follows from the fact that the double heterozygotes, which are selectively favoured, preserve equal numbers of A^R^ and B^R^ genes and this number is a larger proportion of whichever gene is rarer*”

The first statement appears not to apply in our parameter ranges as PRCC and classification trees suggested dominance is not a key determinant of the optimal strategy. The second quote has been extremely influential in guiding deployment of new insecticides. Many key mosquito vector species have already evolved resistance to most of the existing insecticides [2], but, hopefully, resistance is not yet present to the new insecticide. A mismatch in initial gene frequencies will therefore be inevitable, with resistance to the “new” insecticide being lower than that to the old insecticides. Hence Curtis’s statement could be interpreted as suggesting that deploying a new insecticide as a mixture with an existing insecticide would be counter-productive for the new insecticide because resistance to the new insecticide would spread faster if used in a mixture compared to if it was used alone. However there are two way of interpreting Curtis’s statement that differ depending on how the key phrase “more rapid increase” is interpreted. The first interpretation, which appears to be widely held (pers. comms.), assumes that this phrase indicates a comparison is being made between using the “new” insecticide on its own compared to the “new” insecticide being deployed as a mixture with an existing insecticide to which resistance has already spread. This appears to be the most obvious interpretation given the context, but it has two drawbacks. First, it conflicts with our results (which appear consistent in every other aspect). The increase in frequency of resistance to the new insecticide over a single generation when used as a mixture (i.e. ‘B’, the one at lower frequency) is from 0.1% to 0.118% (data extracted from Table 1, example iv in [17]). Curtis does not provide analogous increases for sequential use, but applying Equation S3 in the Supporting Information gives the increase in frequency when then insecticide is deployed on its own as rising from 0.1% to 0.98%. This value of 0.98% is also obtained when running our full IR model so it appears that this interpretation is not supported by the data. Secondly, Curtis did not provide the data to justify this comparison which would, in our opinion, be uncharacteristic of him. The second interpretation is that the key phrase “more rapid increase” refers to a comparison between the lower-frequency and the higher-frequency alleles when insecticides are used in the same mixture, i.e. that the increase in frequency of the low-frequency allele would be larger than the higher-frequency allele. This interpretation seems less likely given the context, but has two points in its favour: it is consistent with our results, and Curtis provides the data to justify the statement. The lower-frequency allele increases from 0.1% to 0.118% i.e. by a factor of 1.18, while the higher-frequency resistance increases from 1% to 1.108 i.e. by a factor of 1.108 (both sets of data are given on Table 1 in [17]). We shall never know definitively which interpretation is correct (Curtis is deceased) but would urge caution in interpreting this second statement from Curtis.

The impact of this re-analysis of Curtis’s results for insecticide deployment strategy depends on the context. If a single new insecticide is available, and we no longer care about maintaining the effectiveness of pre-existing insecticides, it is always optimal (if we ignore the increased cost) to deploy the new insecticide as a mixture with pre-existing ones because IR always spreads slower to new insecticides deployed as a mixture compared to deployed alone. If we do care about pre-existing insecticides, for example pyrethroids, then a decision has to made that maximises the duration of effectiveness for both insecticides and that decision depends on a variety of factors (Figs 7 to 10). Alternately, a decision may be taken to delay deploying the new insecticide until a second new insecticide has become available so that a mixture can be deployed that contains two new insecticides with each, hopefully, having very low resistance levels in the mosquito populations.

Moving on from Curtis, we investigated a wider range of parameter space. A simple analysis based on the two main parameters identified in the PRCC analysis and decision tree allowed a relatively clear separation of parameter space into those combinations that favoured mixtures vs sequential deployment (Figs 9 and 10); presumably, principle component analysis would provide an even clearer separation but would pay a heavy price in their axes not being easy to interpret. This empirical approach is not guaranteed always to work, but did so here. It shows that mixtures are favoured when the effectiveness of insecticides are higher; presumably this will be the case with new insecticides currently under development so it may well be worthwhile considering their deployment as mixtures.

We emphasise that the timeframe in mosquito generations is arbitrary. It would be over-interpreting our results to infer that if, for example, we predict a mixture would last 200 generations, then it would last 20 years (assuming 10 mosquito generations per year). The timescale is arbitrary and was used solely to compare strategies (mixtures vs sequential) on the same scale rather than to make absolute predictions of times until resistance. Most obviously, time to resistance depends critically on the frequency of resistance at the time insecticides are first deployed and this is generally unknowable.

We used classification trees in our sensitivity analysis because the methodology developed here is primarily designed to identify optimal strategy choice and hence should be made as accessible as possible to decision makers with little (or no) technical interest in modelling. The idea is that decision makers can specify, or commission, the likely parameter ranges and distribution (cf our Table 4) and modellers can return a classification tree that allows decision makers to reach, and justify, a transparent decision. There are two caveats to this approach. The first is to emphasise that decision trees are not independent of parameter space. For example, the level of insecticide effectivenesses are the primary determinant of whether a mixture or sequential strategy is favoured in the tree shown Fig 8. However these variable were varied over a range of 0.3 to 1.0 (Table 4) so, conversely, if we varied their values between 0.3 and 0.35 it is possible that they would have no impact in determining whether mixtures are favoured over sequential use. Similarly, it is important to avoid the interpretation that the “best” strategy is that which is favoured in the majority of the simulations as this also depends on the parameter space being investigated. The second caveat is more subjective. We periodically constructed classification trees during the course of the project and noted that their structure could change markedly as a consequence of even quite small changes in parameter values and/or their distribution. We would counsel users to make some small changes to their parameter space to make informal checks that the decision tree structure is stable. One strategy to increase tree stability would be to prune the tree based on cross validation error as described in the methods but in our parameter space this resulted in a tree whose decision structure was based only on insecticide effectiveness; this is a neat result but lacks the illustrative advantages of the unpruned trees we show on Fig 8.

Similar 2-locus models have been developed before e.g. by Curtis [17] and by Gould [11], Mani [16] and Roush [13] for use in agricultural genetics. The approach here has been to construct a flexible model that will remove the need for each new researcher to re-derive and re-programme the model. The flexibility lies in how the niche exposures are defined and how previous analyses may be run as sub-sets of the whole model with different calibrations, e.g. previous analyses which may have assumed fitness costs to be negligible, assumed complete dominance, and so on. Previous models have focused on paradigm situations and often ignored the operational problems that will arise in practice when attempting to implement these policies. These problems are particularly acute in resource- and infrastructure-poor regions of sub-Saharan Africa. For example, it may well be that a mixture strategy is suggested with a pyrethroid on bednets and carbamate on house walls; the mixture comes from the idea that (most) females will contact both the bednet and house wall in her feeding cycle. However its seems extremely likely that practicalities will result in some house walls not being sprayed with carbamate, or re-spraying being so infrequent that it is often effectively absent. In additional, non public-health insecticide use may play a role in driving resistance, such as agricultural and casual peri-household use of pyrethroids. These uses are outside control of the formal health sector and their deployment is often ad-hoc with little regard for the appropriate concentration being deployed. The reason we set up the niches in the models was to allow us to investigate how deviations from the paradigm, recommended implementations may undermine the benefits of that strategy. It may be that even small departures from the paradigm may have major consequences. In the similar case of using drug combinations to treat malaria, it was realised early in the modelling processes that the benefits of combination therapy decline sharply if parasites resistant to one drug had even a small chance of surviving treatment with the combination [27]. One point to note is that operational failings alter the distribution of mosquitoes across niches (Table 1) and not their finesses within these niches (Table 2; the latter reflect insect physiology), which makes the impact of operationally shortfalls relatively easy to investigate within this methodology. The model described here is designed to be highly flexible. We have used upper case ‘A’ and ‘B’ to indicate high insecticide concentrations as opposed to the lower concentrations indicated by their lower case equivalents. It is simple, and valid, to simply change their definition so that, for example, lower case may mean exposure to larvicides in breeding sites, while upper case indicates exposure in adults. The ‘niche’ structure also allows more specific strategy decisions to be explored using more specific calibrations.

The main policies suggested for vector control are sequential use, mixtures, and mosaics. We would add rotation as a possible strategy for completeness. Rotations requires the insecticide be periodically rotated irrespective of resistance status; it is widely used in agriculture of developed countries where infrastructure, experience and expertise is sufficiency well developed to allow its implementation and commercial penalties or incentives may be applied to ensure adherence to deployment recommendations. We would also split mosaics into two types. “Micro-mosaics” which occur over small geographic scales: examples would be houses in a village sprayed with different insecticides, or if children are given bednets that use a different insecticide from adults. In this case we can assume a single, randomly mating mosquito population that can be investigated simply by using two of the single-insecticide niches in Table 1 in conjunction with the unexposed “-, -”niche. “Macro-mosaics” occur over larger geographic scales for example different villages or even health districts may use different insecticides. This geographic separation may invalidate the assumption of random mating within a single mosquito population. The appropriate strategy would then be to run two (or more) versions of the above model, each model corresponding to one piece of the mosaic. Mosquito movement, and hence gene flow, between mosaic “patches” can then be incorporated by allowing a certain proportion of gametes to be exchanged between the models in each generation.

One of the main drawbacks of previous models, at least in our opinions, is that insecticide deployment is assumed to be ‘perfect’, i.e. mixtures are always deployed as mixtures (rather than occasionally as single insecticides when the other is unobtainable). We therefore included an illustrative “correct deployment” parameter. This was meant to demonstrate ‘proof-of-principle’ rather than being definitive and there are important points to be made about its construction. Firstly, this effect may bias analyses against mixtures because sequential, single-insecticide use was assumed to be unaffected by poor deployment which would presumably be manifested by their deployment at concentrations lower than those recommended. The reason we did not have poor adherence to the latter is that its means “A” and “B” niches would be reduced to “a” and “b” respectively, which means we need a whole suite of parametrisation for low insecticide levels. Poor deployment of mixtures is also assumed to be symmetrical i.e. the recommended “AB” is equally likely to go to “A” and “B” whereas in reality, the more expensive (or locally saleable) insecticide may more frequently be absent from the mixture. Finally, the poor deployment pattern is constant over the whole simulation time whereas in reality poor deployment is more likely to occur as random fluctuations over time depending on temporal problems in insecticide supply chain management. In summary, the effect of poor deployment is included here as a simple proof-of-principle demonstration, capable of being elaborated and refined in models that investigate more specific deployment scenarios.

We have focused on genetic arguments in this manuscript (i.e., which strategy minimises the spread of resistance) and have given little consideration to operational implications e.g. the additional cost of mixtures, possible additional impacts of mixtures on non-vector species in the environment, commercial considerations and risk. For example, when there are only two or three insecticides then combining them in a mixture is risking everything if that mixture fails rapidly. Three novel insecticides are being developed through the IVCC and it may be commercially difficult to keep products off-market while they run through a sequence awaiting resistance to evolve. Such considerations could be added to our modelling framework but we have avoided doing so, in the interest of simplicity and brevity. Here we simply recognised the unpredictability of their cost/impact by considering a decision based on the need of mixtures to last >20% longer than sequential to offset these uncertainties.

The option to allow a locus to be sex-linked was included in the model because *Anopheles gambiae* has only 2 autosomes and one sex chromosome. In contrast other vectors, notable *Aedes aegypti*, lack distinct sex chromosomes and have single male-determining loci on otherwise “normal” autosomes. The X chromosome in *A*. *gambiae* is relatively small and heterochromatic in some regions but appears to contains 8.2% of the known coding genes (information extracted from the VectorBase, *Anopheles gambia* page at https://www.vectorbase.org/Anopheles_gambiae/Location/Genome; the X encodes 1,068 out of 13,007 known coding genes) so it is unclear how likely it is that IR-encoding mutations would arise on the X chromosome. The know main IR-mutations in *A*. *gambiae*, ace-1 and kdr, are autosomal although there are reports that X-linked selective sweeps have contributed to IR [29]. In any case, the ability to investigate the potential role of sex-linked genes to IR is an important potential use of the methodology described above. Another reason for having this option is that many types of genetic constructs intended for release into, and control of, mosquito populations (recently reviewed in [30]) attempt to manipulate adult sex-ratio and it is entirely plausible that suppressors of such constructs will primary arise on the X chromosome most obviously in the “X-shredder” construct.

There was a flurry of theoretical work in the 1980s investigating whether to use sequential applications or mixtures of insecticides. Space precludes a full discussion of this work, but can be accessed by reviews such as those by Tabashnik [14] and Roush [31]. However some works did explicitly apply 2-locus models and it is important to reconcile their results with those presented here. The paper of Curtis (1985) is extensively discussed above. Mani (1985) in a highly cited paper (153 citations to date) concluded that “It is shown that the use of mixtures is always more effective in delaying the onset of resistance, often by many orders of magnitude.” This contrasts sharply with our conclusions (and those of Gould and Roush, see below) that sequential use may be favoured in a significant number of scenarios. The differences probably arose from the parameter space examined by Mani in his simulations. He assumed SS genotypes are always killed by the insecticide (i.e. “insecticide effectiveness” = 1 in our terminology), while RR genotypes always survived. He also did not vary exposure but used an “escape” parameter that was fixed at 0.1. Thus, the two factors that we found most important for predicting whether or not sequential use or mixtures were favoured i.e. insecticide effectiveness and exposure (e.g. our Figs 7 to 10) were not varied in his model and were set at values that our results show would greatly favour the use of mixtures (our Fig 9). Gould (1986; [11]) and Roush (1998; [13]) investigated insecticidal toxins genetically-engineered into plants rather than externally-applied insecticides. The evolution of resistance in these contexts are analogous. Gould [11] assumed additive fitness effects (rather than multiplicative as used here) and assumed mortality of SS homozygotes to be 24% to 48%. He did differ from other work by explicitly considering the impact of resistance on control, see discussion below around Eq 9. The ‘insecticide’ was switched in a sequence when the fitness of the mosquito population fell below 0.8 and this level also served as the definition of when control ceased (equivalent to our time to resistance) when comparing the longevity of different strategies (these criteria differ from those of resistance allele frequency used here and in publications such as Curtis [17]). He examined a wide parameter space and concluded that no single method was clearly superior; this conclusion is obviously highly compatible with those presented here. Roush [13] was more definite: he identified mortality of SS homozygotes (our “insecticide effectiveness” parameter) as critical (his Fig 3) and concluded (page 1784) that “As a result of incomplete coverage and residue decay, the mortality of susceptible homozygotes is rarely consistently high enough for pesticide mixtures to be effective”; our sensitivity analyses supports this assertion, and the choice between sequential and mixture deployment appears to rest mainly on how confident we are that the insecticides will reliably and consistently kill the SS homozygotes.

One question, often raised in meetings, is why not routinely use insecticides as mixtures given that drug combinations are now mandatory for treating many infections including the big three global killers: malaria, tuberculosis and HIV/Aids? The use of drug combinations for TB and HIV were driven by clinical observations that resistance almost inevitably arose in patients given monotherapies. However the dynamics of how resistance arises in these two infections are very different to how insecticide resistance arises. Resistance in TB and HIV occur mainly by within-host dynamics: new, spontaneous mutations arise in the huge number of individual pathogens present in that patient and then spread within-patient to dominate the infection. Also, (crudely speaking; see [32]), there are no refugia or differential drug exposure within a patient, and patients may be dosed so that both/all drugs in mixture are fully effective against sensitive pathogens. Malaria is somewhat different, and more similar to insecticide resistance, in that drug-resistant mutations arise relatively rarely and then spread throughout the population (although this depends on the drug: resistance to atovaquone arises very easily from within the infection but, interestingly, has a lethal fitness cost in the insect vector [33]). The use of drug combinations to treat malaria was therefore predicted on the type of population genetic models described above for insecticide resistance (see [6] for access to the literature) which showed that drug combinations should slow the input and subsequent spread of drug-resistant mutations. These early genetic models tended to assume the antimalarial drugs were fully effective against sensitive parasites and/or that the double-resistant genotypes were completely unaffected by the drugs (these genotypes had resistance alleles at two loci; malaria is haploid so has no heterozygotes). The one model that did allow relatively low selection pressures against malaria parasites with both resistant and sensitive alleles [27] had selection pressures that depended on “the fraction of infected persons who are treated, dosage, and so on”; in our terminology this confounded exposure and effectiveness. It will therefore be highly informative to revisit these models of antimalarial drug resistance and investigate how robust they are when the assumption of complete drug effectiveness is removed; we are currently undertaking these analyses.

## Future Directions

The results presented above use only a part of the functionality that was built into our model to make it as flexible as possible. Many simplifications made by us have been noted in the previous literature. For example, Tabashnik [15] discussed the impact of mutations encoding cross-resistance to both insecticides and noted that mixtures (and presumably mosaics) would constitute “intense selection for cross resistance”; this cross resistance can be incorporated using the antagonist/synergistic functions on our S1 Table. Tabashnik [15] also noted that “an implicit assumption of all these models is that the pesticides in the mixture have equal persistence so that no individuals are exposed to only one pesticide” (page 1264): again this is simple to incorporate by using AB as the niche encountered when both insecticides are present at high concentrations, Ab when the faster degrading insecticide, B/b in this case, is at relatively low concentrations, and a, - when insecticide A has fallen to low concentrations and b has fallen to ineffective concentration. We also note that policies are changed and insecticides deemed “ineffective” when resistance allele frequency reaches 50%; this was done to maintain consistency with previous work. This criterion presupposes that the resistance allele has been identified so that its frequency can be tracked. The 50% allele frequency also has only a weak link with effectiveness; once allele frequency reaches 50%, then if the allele is dominant then 75% of mosquitoes will have resistant genotypes (i.e. RS, RR), whereas if it is recessive only 25% of mosquitoes are resistant (RR). Moreover, these resistant genotypes may differ in their fitness so that even the RR forms are often killed by contact. The methodology allows a more flexible definition of when a switch occurs and/or when an insecticide becomes ineffective (e.g. >25% of mosquitoes surviving exposure); again, this is straightforward to code (see Eq 9 below) and should be addressed in the future.

One other factor, not considered in our model, is that mixture may kill more mosquitoes than single use. As a simple example, if resistance allele frequency has reached the level where 40% of mosquitoes survive contact if used alone. If used in a mixture where the other insecticide kills 30% of the population, then the proportion surviving the mixture will be 40% x 30% = 12% i.e., the mixture will have a much higher killing rate than either insecticide used alone and will, putatively, have a greater impact on disease transmission. This raises another, final, point. Population genetic models such as the one developed here only track the frequencies of genotypes in the populations. They do not predict how this impacts the population size or female longevity of the mosquito population which are key determinants of disease transmission if the mosquito is a vector (reviewed in [34]). We can go some way to addressing this by calculating the relative fitness of the population being controlled (really the reduction in egg lay) compared to a fully sensitive population in the absence of insecticide following Gould [11] as

$$\bar{W}=\overset{10}{\underset{i=1}{\sum }}F^{f,i}W^{f,i}$$ (9)

Where summation over i represented the fitnesses of the 10 possible 2-locus genotypes (cf S1 Table). Note that we use females in this summation as we assume sufficient males survive to ensure all eggs are fertilised. How this reduction in egg lay translates into vector population size the next generation is not defined here and it depends on the ecology of the organism i.e. the extent to which density-dependent population regulation helps restore the original population size. Nor does this consider the longevity of mosquitoes which is an important component of vector-borne disease epidemiology. The three disciplines of genetics, ecology and epidemiology are highly interlinked and future modelling should try to build an explicit bridge between the two approaches to investigate specific control methods of specific disease vectors. The work described here builds the foundation for one of these three components.

Importantly, we are not at this point advocating mixtures or sequential deployment as a universal policy measure, simply demonstrating how more modern and powerful modelling techniques may be applied to the research questions raised by Curtis. Our results indicate that the relative performance of mixture and sequential strategies depends upon attributes of the insecticides, their application, mosquito behaviour and genetics. One highly encouraging result is that choosing between mixtures and sequential deployment depends mainly on two parameters, the effectiveness of the insecticides against the SS genotypes and exposure, both of which are under operator control. They can also be measured or predicted; for example experimental huts (e.g. [35–38]) could be used to estimate the effectiveness of insecticides against the SS genotypes. The key point is that we do not need to know anything about the resistant SR and RR genotypes (fitness, dominance) which would be impossible to predict prior to them emerging in the population after the strategy decision has been made.

One practical problem is that deployment strategies will most probably be made on a regional, possibly national, level, and there may well be considerable heterogeneity within the region/country, some areas favouring sequential use and some mixtures. In short the decision will be difficult and will require more directed modelling incorporating the specific attributes of the insecticides, their proposed mode of deployment, and the biological parameters and biometrics of the vector species operating in the proposed deployment area. Hence we have focused here of describing the requisite methodology and postpone detailed investigation of specific scenarios to future publications.

## Supporting Information

S1 Table Finesses of two-locus genotypes in the 9 environmental niches. The niches are defined as in Table 1 of the main text, w is fitness of the individual one-locus genotypes as defined in Table 2 of the main text, and Λ parameter represent the effect of antagonism/synergism/cross-resistance in niches where mosquitoes encountered both insecticides. The symbols (c) and (r) in the genotypes indicate whether the double heterozygotes are in coupling or repulsion, respectively.
(DOCX)

S2 Table Gamete production from male parental genotypes. The output from females parents is calculated in the same way except that the superscript ‘m’ in the symbols below is replaced by ‘f’. The Mendelian output of the four gamete types from each parental genotype are indicted in bold where r is the recombination rate between the loci. The symbols (c) and (r) in the genotypes indicate whether the double heterozygotes are in coupling or repulsion, respectively.
(DOCX)

S3 Table Diploid progeny formed from fusion of gametes assuming both loci are autosomal. (DOCX)
