# Supplementary material for: A Two-Locus Model of the Evolution of Insecticide Resistance to Inform and Optimise Public Health Insecticide Deployment Strategies
Source: PLoS Comput Biol. 2017 Jan 17;13(1):e1005327. doi: 10.1371/journal.pcbi.1005327 (PMC5283767; doi:10.1371/journal.pcbi.1005327)
Supplement: S1 Table — The niches are defined as in Table 1 of the main text, w is fitness of the individual one-locus genotypes as defined in Table 2 of the main text, and Λ parameter represent the effect of antagonism/synergism/cross-resistance in niches where mosquitoes encountered both insecticides. The symbols (c) and (r) in the genotypes indicate whether the double heterozygotes are in coupling or repulsion, respectively. (DOCX) [file pcbi.1005327.s001.docx]

Table S1. Finesses of two-locus genotypes in the 9 environmental niches. The niches are defined as in Table 1 of the main text, w is fitness of the individual one-locus genotypes as defined in Table 2 of the main text, and Λ parameter represent the effect of antagonism/synergism/cross-resistance in niches where mosquitoes encountered both insecticides. The symbols (c) and (r) in the genotypes indicate whether the double heterozygotes are in coupling or repulsion, respectively.

| **Genotype** | |  | **Insecticide Niche** | | | | | | | | |  |
| --- | --- | --- | --- | --- | --- | --- | --- | --- | --- | --- | --- | --- |
| **Locus1** | **Locus 2** | *-,-* | | *a,-* | *A,-* | *-,b* | *-,B* | *a,b* | *A,b* | *a,B* | *A,B* | |
| *SS* | *SS* | $w_{-}^{SS1}w_{-}^{SS2}$ | | $w_{a}^{SS1}w_{-}^{SS2}$ | $w_{A}^{SS1}w_{-}^{SS2}$ | $w_{-}^{SS1}w_{b}^{SS2}$ | $w_{-}^{SS1}w_{B}^{SS2}$ | $w_{a}^{SS1}w_{b}^{SS2}$ *Λ_ab_ | $w_{A}^{SS1}w_{b}^{SS2}$ *Λ_Ab_ | $w_{a}^{SS1}w_{B}^{SS2}$ *Λ_aB_ | $w_{A}^{SS1}w_{B}^{SS2}$ *Λ_AB_ | |
| *SS* | *RS* | $w_{-}^{SS1}w_{-}^{RS2}$ | | $w_{a}^{SS1}w_{-}^{RS2}$ | $w_{A}^{SS1}w_{-}^{RS2}$ | $w_{-}^{SS1}w_{b}^{RS2}$ | $w_{-}^{SS1}w_{B}^{RS2}$ | $w_{a}^{SS1}w_{b}^{RS2}$ *Λ_ab_ | $w_{A}^{SS1}w_{b}^{RS2}$ *Λ_Ab_ | $w_{a}^{SS1}w_{B}^{RS2}$ *Λ_aB_ | $w_{A}^{SS1}w_{B}^{RS2}$ *Λ_AB_ | |
| *SS* | *RR* | $w_{-}^{SS1}w_{-}^{RR2}$ | | $w_{a}^{SS1}w_{-}^{RR2}$ | $w_{A}^{SS1}w_{-}^{RR2}$ | $w_{-}^{SS1}w_{b}^{RR2}$ | $w_{-}^{SS1}w_{B}^{RR2}$ | $w_{a}^{SS1}w_{b}^{RR2}$ *Λ_ab_ | $w_{A}^{SS1}w_{b}^{RR2}$ *Λ_Ab_ | $w_{a}^{SS1}w_{B}^{RR2}$ *Λ_aB_ | $w_{A}^{SS1}w_{B}^{RR2}$ *Λ_AB_ | |
| *RS* | *SS* | $w_{-}^{RS1}w_{-}^{SS2}$ | | $w_{a}^{RS1}w_{-}^{SS2}$ | $w_{A}^{RS1}w_{-}^{SS2}$ | $w_{-}^{RS1}w_{b}^{SS2}$ | $w_{-}^{RS1}w_{B}^{SS2}$ | $w_{a}^{RS1}w_{b}^{SS2}$ *Λ_ab_ | $w_{A}^{RS1}w_{b}^{SS2}$ *Λ_Ab_ | $w_{a}^{RS1}w_{B}^{SS2}$ *Λ_aB_ | $w_{A}^{RS1}w_{B}^{SS2}$ *Λ_AB_ | |
| *RS* | *RS (c)* | $w_{-}^{RS1}w_{-}^{RS2}$ | | $w_{a}^{RS1}w_{-}^{RS2}$ | $w_{A}^{RS1}w_{-}^{RS2}$ | $w_{-}^{RS1}w_{b}^{RS2}$ | $w_{-}^{RS1}w_{B}^{RS2}$ | $w_{a}^{RS1}w_{b}^{RS2}$ *Λ_ab_ | $w_{A}^{RS1}w_{b}^{RS2}$ *Λ_Ab_ | $w_{a}^{RS1}w_{B}^{RS2}$ *Λ_aB_ | $w_{A}^{RS1}w_{B}^{RS2}$ *Λ_AB_ | |
| *RS* | *RS (r)* | $w_{-}^{RS1}w_{-}^{RS2}$ | | $w_{a}^{RS1}w_{-}^{RS2}$ | $w_{A}^{RS1}w_{-}^{RS2}$ | $w_{-}^{RS1}w_{b}^{RS2}$ | $w_{-}^{RS1}w_{B}^{RS2}$ | $w_{a}^{RS1}w_{b}^{RS2}$ *Λ_ab_ | $w_{A}^{RS1}w_{b}^{RS2}$ *Λ_Ab_ | $w_{a}^{RS1}w_{B}^{RS2}$ *Λ_aB_ | $w_{A}^{RS1}w_{B}^{RS2}$ *Λ_AB_ | |
| *RS* | *RR* | $w_{-}^{RS1}w_{-}^{RR2}$ | | $w_{a}^{RS1}w_{-}^{RR2}$ | $w_{A}^{RS1}w_{-}^{RR2}$ | $w_{-}^{RS1}w_{b}^{RR2}$ | $w_{-}^{RS1}w_{B}^{RR2}$ | $w_{a}^{RS1}w_{b}^{RR2}$ *Λ_ab_ | $w_{A}^{RS1}w_{b}^{RR2}$ *Λ_Ab_ | $w_{a}^{RS1}w_{B}^{RR2}$ *Λ_aB_ | $w_{A}^{RS1}w_{B}^{RR2}$ *Λ_AB_ | |
| *RR* | *SS* | $w_{-}^{RR1}w_{-}^{SS2}$ | | $w_{a}^{RR1}w_{-}^{SS2}$ | $w_{A}^{RR1}w_{-}^{SS2}$ | $w_{-}^{RR1}w_{b}^{SS2}$ | $w_{-}^{RR1}w_{B}^{SS2}$ | $w_{a}^{RR1}w_{b}^{SS2}$ *Λ_ab_ | $w_{A}^{RR1}w_{b}^{SS2}$ *Λ_Ab_ | $w_{a}^{RR1}w_{B}^{SS2}$ *Λ_aB_ | $w_{A}^{RR1}w_{B}^{SS2}$ *Λ_AB_ | |
| *RR* | *RS* | $w_{-}^{RR1}w_{-}^{RS2}$ | | $w_{a}^{RR1}w_{-}^{RS2}$ | $w_{A}^{RR1}w_{-}^{RS2}$ | $w_{-}^{RR1}w_{b}^{RS2}$ | $w_{-}^{RR1}w_{B}^{RS2}$ | $w_{a}^{RR1}w_{b}^{RS2}$ *Λ_ab_ | $w_{A}^{RR1}w_{b}^{RS2}$  *Λ_Ab_ | $w_{a}^{RR1}w_{B}^{RS2}$ *Λ_aB_ | $w_{A}^{RR1}w_{B}^{RS2}$ *Λ_AB_ | |
| *RR* | *RR* | $w_{-}^{RR1}w_{-}^{RR2}$ | | $w_{a}^{RR1}w_{-}^{RR2}$ | $w_{A}^{RR1}w_{-}^{RR2}$ | $w_{-}^{RR1}w_{b}^{RR2}$ | $w_{-}^{RR1}w_{B}^{RR2}$ | $w_{a}^{RR1}w_{b}^{RR2}$ *Λ_ab_ | $w_{A}^{RR1}w_{b}^{RR2}$ *Λ_Ab_ | $w_{a}^{RR1}w_{B}^{RR2}$ *Λ_aB_ | $w_{A}^{RR1}w_{B}^{RR2}$ *Λ_AB_ | |
