# Supplementary material for: A Two-Locus Model of the Evolution of Insecticide Resistance to Inform and Optimise Public Health Insecticide Deployment Strategies
Source: PLoS Comput Biol. 2017 Jan 17;13(1):e1005327. doi: 10.1371/journal.pcbi.1005327 (PMC5283767; doi:10.1371/journal.pcbi.1005327)
Supplement: S2 Table — The output from females parents is calculated in the same way except that the superscript ‘m’ in the symbols below is replaced by ‘f’. The Mendelian output of the four gamete types from each parental genotype are indicted in bold where r is the recombination rate between the loci. The symbols (c) and (r) in the genotypes indicate whether the double heterozygotes are in coupling or repulsion, respectively. (DOCX) [file pcbi.1005327.s002.docx]

**Table S2.**  Gamete production from male parental genotypes; the output from females parents is calculated in the same way except that the superscript ‘m’ in the symbols below is replaced by ‘f’. The Mendelian output of the four gamete types from each parental genotype are indicted in bold where r is the recombination rate between the loci. The symbols (c) and (r) in the genotypes indicate whether the double heterozygotes are in coupling or repulsion, respectively.

| Parental genotype | | Gamete haplotype (allele at locus 1 is given first) | | | |
| --- | --- | --- | --- | --- | --- |
| **Locus 1** | **Locus 2** | SS | RS | SR | RR |
| *SS* | *SS* | F^m,SS1SS2^ * W^m,SS1SS2^ ***1** | *F^m,SS1SS2^* * *W^m,SS1SS2^* ***0** | *F^m,SS1SS2^* * *W^m,SS1SS2^* ***0** | *F^m,SS1SS2^* * *W^m,SS1SS2^* ***0** |
| *SS* | *RS* | F^m,SS1RS2^ * W^m,SS1RS2^ ***0.5** | F^m,SS1RS2^ * W^m,SS1RS2^ ***0** | F^m,SS1RS2^ * W^m,SS1RS2^***0.5** | F^m,SS1RS2^ * W^m,SS1RS2^***0** |
| *SS* | *RR* | F^m,SS1RR2^ * W^m,SS1RR2^ ***0** | F^m,SS1RR2^ * W^m,SS1RR2^ ***0** | F^m,SS1RR2^ * W^m,SS1RR2^ ***1** | F^m,SS1RR2^ * W^m,SS1RR2^ ***0** |
| *RS* | *SS* | F^m,RS1SS2^ * W^m,RS1SS2^ ***0.5** | F^m,RS1SS2^ * W^m,RS1SS2^ ***0.5** | F^m,RS1SS2^ * W^m,RS1SS2^ ***0** | F^m,RS1SS2^ * W^m,RS1SS2^ ***0** |
| *RS* | *RS (c)* | F^m,RS1RS2(c)^ * W^m,RS1RS2^ ***(1-r)/2** | *F^m,RS1RS2(c)^* * *W^m,RS1RS2^* ***(r/2)** | *F^m,RS1RS2(c)^* * *W^m,RS1RS2^* ***(r/2)** | *F^m,RS1RS2(c)^* * *W^m,RS1RS2^* ***(1-r)/2** |
| *RS* | *RS (r)* | F^m,RS1RS(r)^ * W^m,RS1RS2^ ***(r/2**) | *F^m,RS1RS(r)^* * *W^m,RS1RS2^* ***(1-r)/2** | *F^m,RS1RS(r)^* * *W^m,RS1RS2^* ***(1-r)/2** | *F^m,RS1RS(r)^* * *W^m,RS1RS2^* ***(r/2**) |
| *RS* | *RR* | F^m,RS1RR2^ * W^m,RS1RR2^ ***0** | F^m,RS1RR2^ * W^m,RS1RR2^ ***0** | F^m,RS1RR2^ * W^m,RS1RR2^ ***0.5** | F^m,RS1RR2^ * W^m,RS1RR2^ ***0.5** |
| *RR* | *SS* | F^m,RR1SS2^ * W^m,RR1SS2^ ***0** | F^m,RR1SS2^ * W^m,RR1SS2^ ***1.0** | F^m,RR1SS2^ * W^m,RR1SS2^ ***0** | F^m,RR1SS2^ * W^m,RR1SS2^ ***0** |
| *RR* | *RS* | F^m,RR1RS2^ * W^m,RR1RS2^ ***0** | F^m,RR1RS2^ * W^m,RR1RS2^ ***0.5** | F^m,RR1RS2^ * W^m,RR1RS2^ ***0** | F^m,RR1RS2^ * W^m,RR1RS2^ ***0.5** |
