# Supplementary material for: A Two-Locus Model of the Evolution of Insecticide Resistance to Inform and Optimise Public Health Insecticide Deployment Strategies
Source: PLoS Comput Biol. 2017 Jan 17;13(1):e1005327. doi: 10.1371/journal.pcbi.1005327 (PMC5283767; doi:10.1371/journal.pcbi.1005327)
Supplement: S3 Table — (DOCX) [file pcbi.1005327.s003.docx]

**Table S3.** Diploid progeny formed from fusion of gametes assuming both loci are autosomal.

| Maternal gamete -> | $G_{f}^{SS}$ | | | | $G_{f}^{SR}$ | | | | $G_{f}^{RS}$ | | | | $G_{f}^{RR}$ | | | |
| --- | --- | --- | --- | --- | --- | --- | --- | --- | --- | --- | --- | --- | --- | --- | --- | --- |
| Paternal gamete -> | $G_{m}^{SS}$ | $G_{m}^{SR}$ | $G_{m}^{RS}$ | $G_{m}^{RR}$ | $G_{m}^{SS}$ | $G_{m}^{SR}$ | $G_{m}^{RS}$ | $G_{m}^{RR}$ | $G_{m}^{SS}$ | $G_{m}^{SR}$ | $G_{m}^{RS}$ | $G_{m}^{RR}$ | $G_{m}^{SS}$ | $G_{m}^{SR}$ | $G_{m}^{RS}$ | $G_{m}^{RR}$ |
| ***Diploid progeny ↓*** |  |  |  |  |  |  |  |  |  |  |  |  |  |  |  |  |
| SS1SS2 | 1 |  |  |  |  |  |  |  |  |  |  |  |  |  |  |  |
| SS1SR2 |  | 1 |  |  | 1 |  |  |  |  |  |  |  |  |  |  |  |
| SS1RR2 |  |  |  |  |  | 1 |  |  |  |  |  |  |  |  |  |  |
| SR1SS2 |  |  | 1 |  |  |  |  |  | 1 |  |  |  |  |  |  |  |
| SR1SR2(c) |  |  |  | 1 |  |  |  |  |  |  |  |  | 1 |  |  |  |
| SR1SR2(r) |  |  |  |  |  |  | 1 |  |  | 1 |  |  |  |  |  |  |
| SR1RR2 |  |  |  |  |  |  |  | 1 |  |  |  |  |  | 1 |  |  |
| RR1SS2 |  |  |  |  |  |  |  |  |  |  | 1 |  |  |  |  |  |
| RR1SR2 |  |  |  |  |  |  |  |  |  |  |  | 1 |  |  | 1 |  |
| RR1RR2 |  |  |  |  |  |  |  |  |  |  |  |  |  |  |  | 1 |
